# Facilitating the Sharing of Electrophysiology Data Analysis Results Through In-Depth Provenance Capture

**DOI:** 10.1523/ENEURO.0476-23.2024

**Published:** 2024-06-07

**Authors:** Cristiano A. Köhler, Danylo Ulianych, Sonja Grün, Stefan Decker, Michael Denker

**Affiliations:** ^1^Institute for Advanced Simulation (IAS-6) and JARA Institute Brain Structure-Function Relationships (INM-10), Jülich Research Centre, 52428 Jülich, Germany; ^2^Theoretical Systems Neurobiology, RWTH Aachen University, 52062 Aachen, Germany; ^3^Chair of Databases and Information Systems, RWTH Aachen University, 52074 Aachen, Germany; ^4^Fraunhofer Institute for Applied Information Technology (FIT), 53757 Sankt Augustin, Germany

## Abstract

Scientific research demands reproducibility and transparency, particularly in data-intensive fields like electrophysiology. Electrophysiology data are typically analyzed using scripts that generate output files, including figures. Handling these results poses several challenges due to the complexity and iterative nature of the analysis process. These stem from the difficulty to discern the analysis steps, parameters, and data flow from the results, making knowledge transfer and findability challenging in collaborative settings. Provenance information tracks data lineage and processes applied to it, and provenance capture during the execution of an analysis script can address those challenges. We present Alpaca (Automated Lightweight Provenance Capture), a tool that captures fine-grained provenance information with minimal user intervention when running data analysis pipelines implemented in Python scripts. Alpaca records inputs, outputs, and function parameters and structures information according to the W3C PROV standard. We demonstrate the tool using a realistic use case involving multichannel local field potential recordings of a neurophysiological experiment, highlighting how the tool makes result details known in a standardized manner in order to address the challenges of the analysis process. Ultimately, using Alpaca will help to represent results according to the FAIR principles, which will improve research reproducibility and facilitate sharing the results of data analyses.

## Significance Statement

Sharing electrophysiology data analysis results is challenging, especially in collaborative environments. The results can be understood and interpreted only with the accurate description of the individual analysis steps, the parameters, and the data flow, which can be achieved by storing the results together with detailed provenance information. We implemented the Alpaca toolbox to capture provenance during the execution of Python scripts, a typical implementation in pipelines that analyze electrophysiology datasets. Alpaca provides an easy and lightweight solution to record the relevant details of the analysis, facilitating sharing the results.

## Introduction

Electrophysiology methods are routinely used to investigate brain function, including the measurement of extracellular potentials using microelectrodes implanted into brain tissue (Buzsáki et al., 2012; Huang, 2016). The first electrophysiology experiments acquired potentials from single or few implanted electrodes, which limited the data throughput of the experiments. However, recent technological advances produced large-density electrode arrays and data acquisition systems able to record hundreds of channels from heterogeneous sources in the experiment sampled at high resolution (Hong and Lieber, 2019). It is now possible to perform massive and parallel recordings during electrophysiology experiments (Buzsáki, 2004) that result in datasets that are both complex in structure and large in volume.

For the analysis of such datasets, this introduces two major consequences. First, the analysis will often be partially conducted in an exploratory style, where the analysis parameters and selection of datasets are probed iteratively by the scientists. Keeping track of these choices and approaches is particularly challenging for the scientist in the context of complex data. Second, the analysis of modern datasets often requires advanced methods (Brown et al., 2004; Stevenson and Kording, 2011) that are implemented as workflows composed of several interdependent scripts (Denker and Grün, 2016, for a detailed description). The highly diverse and distributed results from the parallel and intertwined processing pipelines operating on complex data must be organized and described in a manner that is comprehensible not only to the original author of the analysis workflow but also in a collaborative context. Taken together, the full workflow including iterative and pipeline approaches, starting from the experimental data acquisition to the presentation of final results, is subject to a hierarchical decision-making process, frequent changes, and a large number of processing steps. With growing complexity, these aspects are increasingly difficult to follow, especially in collaborative contexts, where results of analyses executed by different scientists are shared.

The resulting lack of reproducibility undermines the scientific investigations and the public trust in the scientific method and results (cf., Baker, 2016). In collaborative environments, the details of an executed analysis workflow should not only be fully documented but also readily understandable by all partners. Thus, work in collaboration could be improved further by directly capturing provenance information on a coarser level of granularity that is informative of the data manipulations throughout the execution of an analysis workflow leading to a certain analysis result (Ragan et al., 2016; Pimentel et al., 2019). By using a provenance tracking system during workflow execution, all operations performed on a given data object can be described and stored in an accessible and structured way that is comprehensible to a human. For the analysis of an electrophysiology dataset, those operations consist of specific analysis methods or processes, such as applying a bandpass filter, downsampling a specific recorded signal, or generating a plot. Ultimately, the details relevant for the final interpretation of the results can be captured and, ideally, stored as metadata with the analysis results. These may then represent summaries of the analysis flow and lead to a description of the results that improve findability, interoperability, and reusability of the results (FAIR principles, Wilkinson et al., 2016).

Several tools to track and record provenance within (analysis) workflows and single scripts exist, spanning different domains (Ragan et al., 2016; Pimentel et al., 2019). The tools take different approaches depending on which type of information to capture (e.g., tracing code execution, capturing user interactions, or monitoring operating system calls), and the implementation varies according to the intended use of the captured provenance information and its granularity (Bavoil et al., 2005; MacKenzie-Graham et al., 2008; Köster and Rahmann, 2012; Davison et al., 2014; Murta et al., 2015; Pizzi et al., 2016). Although some of these solutions might be adapted or even combined to use in the analysis of electrophysiology data, none of these are designed and optimized with the particularities of this type of analysis setting in mind. One of these particularities to consider is the ease of use with custom analysis scripts. A workflow management system (WMS) such as *VisTrails* (Bavoil et al., 2005), for instance, requires the construction of workflows from analysis modules implemented as part of the WMS framework or by writing plugins, when the user might need the flexibility of custom scripts. Likewise, a tool such as *AiiDA* (Pizzi et al., 2016) provides a full workflow ecosystem that requires the development of plugins and wrappers to interface and enforces its own data types, hindering the reuse of existing code and libraries without considerable effort. A second aspect to consider is the level of detail and suitability of the provenance information. For individual scripts, tools like *noWorkflow* (Murta et al., 2015) produce a provenance trail that is highly detailed and without semantic meaning, making it difficult for the scientist to extract information. In contrast, a tool like *Sumatra* (Davison et al., 2014) will record a more global context in which a script is run in the command line (script parameters, execution environment, version history, and links to the output files), while specific operations inside the script will not be detailed. Solutions to orchestrate a series of scripts, like *Snakemake* (Köster and Rahmann, 2012), produce flow graphs that show the flow of execution for the scripts composing a workflow but lack the actions performed within each script such that more detailed provenance metadata must be manually recorded by the user without any standardization. Finally, a last aspect is the specificity of the tools for a certain scientific domain. For example, a tool like *LONI Pipeline*, that supports full workflows with provenance tracking for the analysis of neuroimaging data (MacKenzie-Graham et al., 2008), could be readily used in some analysis scenarios. However, the specificity of the available workflow components is a disadvantage for the user that wants to implement pipelines that fall outside of the scope of the intended use.

This work sets out to address the challenges associated with the analysis of an electrophysiology dataset and sharing the results. To accomplish this, a novel tool was implemented to capture the suitable scope of provenance information and store it as metadata together with results generated by analysis scripts implemented in the *Python* programming language. A typical analysis scenario is presented as a use case and then the tool is analyzed with respect to the challenges it aims to address.

## Materials and Methods

### Challenges for provenance capture during the analysis of electrophysiology data

We argue that a tool to capture provenance information during the analysis of electrophysiology data has to deal with four scenarios: (i) the analyses often require several preprocessing steps before any analytical method is applied, (ii) the data analysis process is often not linear but intertwined and therefore exhibits a certain level of intricacy, (iii) parameters of the analysis are frequently and often iteratively probed, and (iv) the final results are likely to be published or used in shared environments. In the following, we describe these scenarios in detail and derive four associated challenges for capturing provenance.

**Figure 1.**
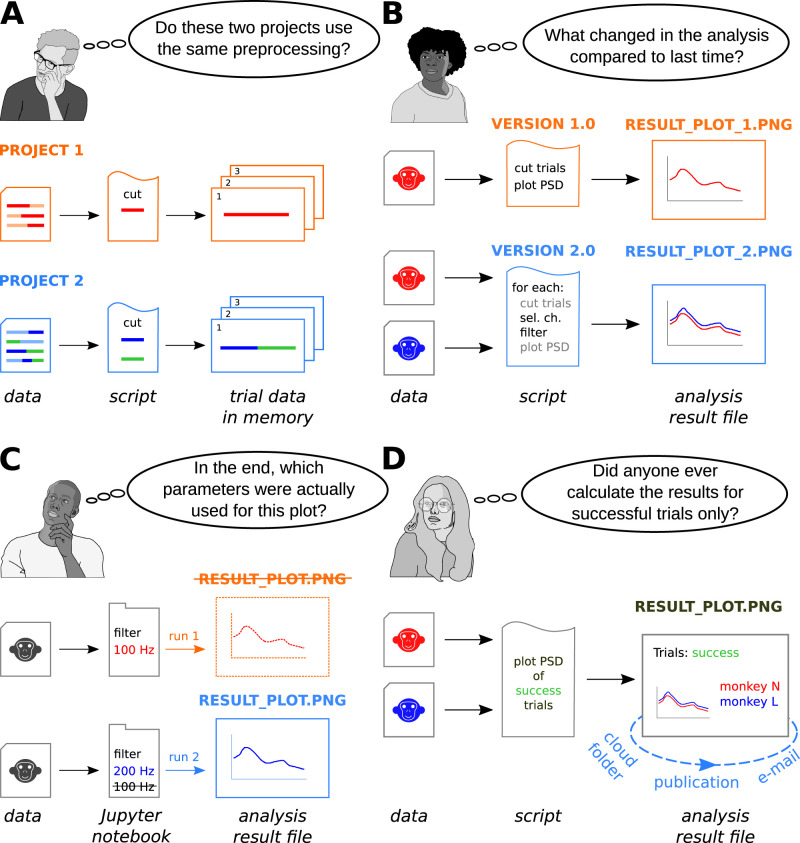
Overview of the four representative scenarios leading to challenges associated with the analysis of an electrophysiology dataset. ***A***, Data frequently require preprocessing before the analytic methods are applied. This step is often customized for each project, resulting in distinct pipelines that are typically implemented on the level of a script for reasons of efficiency. ***B***, The structure of an analysis script is not static throughout the analysis process. It can be updated to accommodate new data or hypotheses. This results in multiple versions of scripts with increasingly complex pipelines, which are associated with distinct versions of the results. ***C***, Relevant parameters for the analyses are often probed iteratively, with the subsequent results changing in time. Keeping track of the exact parameters used for specific results becomes difficult. ***D***, Use of results in shared environments and publishing results requires making available the details of the analysis process to all collaborators. Finding specific results in a shared repository of results from different collaboration partners is difficult, as relevant parameters may be stored in a non-machine-readable format inside the result file, or cryptically in file names.

Preprocessing is a typical step in the analysis, and is usually custom-tailored to a particular project (Fig. 1*A*). For instance, data from a recording session of multiple trials (e.g., repeated stimulus presentations or behavioral responses) are usually recorded as a single data stream and only during the analysis cut into the individual trial epochs relevant to the analysis goal. Due to the high level of heterogeneity in the data, this is frequently achieved using custom scripts, with parameters that are specific to the trial structure and design of the experiment (e.g., selecting only particular trials according to behavioral responses such as reaction time). The scientist’s written documentation, source code, and, in many cases, the data itself would need to be inspected to understand all these steps, e.g., the chunking of the data that was performed before the core analysis. Therefore, a first challenge is to clearly document the processing in an accessible and automated manner and to provide this information as supplement to the analysis output.

The full analysis pipeline from the dataset to a final result artifact is likely not built in one attempt, but instead involves a continuous development (Fig. 1*B*). For instance, as new data are obtained, time series may need to be excluded from analysis and new hypotheses are generated. Therefore, the analysis scripts may be updated to include additional analysis steps, and the resulting code will have increasing complexity. One solution to organize this agile process is to use a WMS (*Snakemake*; Köster and Rahmann, 2012) coupled with a code versioning system such as *git*. For each run, the WMS will provide coarse provenance information, such as the name of the script, environment information, script parameters, and files that were used or generated. The scripts can then be tracked to specific versions knowing the *git* commit history. However, if multiple operations (e.g., cutting data, downsampling, and filtering) are performed inside one script, the actual parameters in each step are possibly not captured as part of the provenance. This is the case where provenance information shows only script parameters passed by command line. The mapping of command line to the actual parameters used by the functions in the script relies on the correct implementation of the code, and any default parameters for the function that are not passed by command line will not be known. Furthermore, it is not possible to inspect each intermediate (in-memory) data object during the execution of the script. Yet, without knowledge of these data operations and the data flow, it becomes challenging to compare results generated by multiple versions of the evolving analysis script, in particular if the code structure of the script changes over time. A solution to this challenge could be to break such complex scripts in several smaller scripts, such that the coarse provenance information of the WMS could be more descriptive of each individual process and intermediate results would be saved to disk (i.e., in our example, separate scripts for cutting, downsampling, and filtering). However, this may be inconvenient and inefficient: resource-intensive operations (e.g., file loading and writing) might be repeated across different scripts, and temporary files would have to be used between the steps, instead of efficiently manipulating data in memory. Moreover, this approach limits the expressiveness and creativity of defining data operations as opposed to the full set of operations offered by the programming language in a single script. Therefore, a second challenge is to efficiently capture the parameters and the data flow associated with the analysis steps of the script.

The parameters that control the final analysis output are frequently probed iteratively (Fig. 1*C*). For example, the scientist performing the analysis could write a *Jupyter* notebook (Kluyver et al., 2016) to find specific frequency cutoffs for a filtering step. In one scenario, code cells of the notebook can be run in arbitrary sequences, with some parameters being changed in the process until a result artifact (e.g., a plot) is saved in a file. In a different scenario, it is possible to generate several versions of a given file by the same notebook, each of which overwrites the previous version. At this point, the scientist performing the analysis might rely on the associated *Jupyter* history or versioning of the notebook/files using *git*. However, the relevant parameters that were used to generate results saved in the last version of the file would be difficult to recall. Ultimately, a detailed documentation by the user or retracing the source code according to an execution history is still required. Therefore, a third challenge is to retain a documentation of the iterative generation of the analysis result that is explicitly and unambiguously linked to the generated result file.

The fourth challenge stems from the situation where results (e.g., plots) are likely to be published or used in collaborative environments (Fig. 1*D*). This includes files uploaded in a manuscript submission, or files deposited in a shared folder or sent between collaborators via email. The interpretation of the stored results depends on the understanding of the analysis details and its relevant parameters by the collaboration partner. Moreover, searching for specific results in a large collection of shared files can be difficult: not all the relevant parameters are recorded in the file name, and are likely stored as non-machine-readable information within the file (e.g., an axis label in a figure). In these situations, analysis provenance stored together with the shared result files as structured and comprehensible metadata should improve information transfer in the collaboration and findability of the results.

### Use case scenario

**Figure 2.**
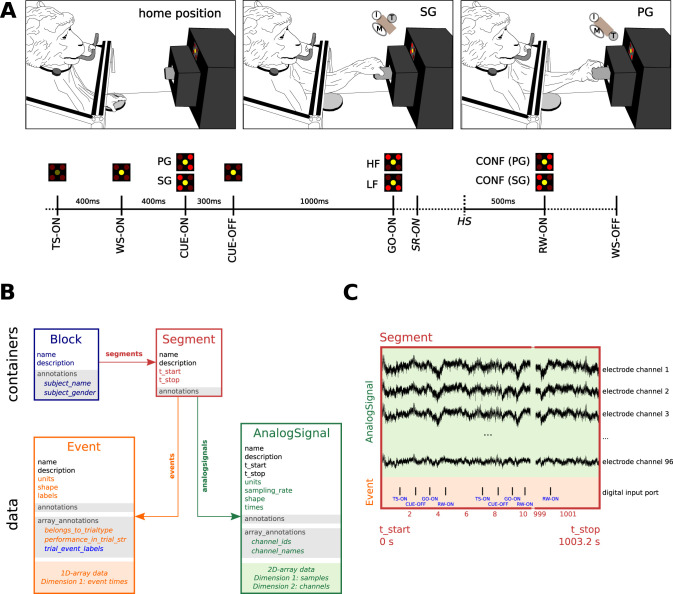
Overview of the delayed reach-to-grasp task and *Neo* data model. The datasets from the experiment were used for use case scenario for capturing provenance during the analysis of electrophysiology data, and *Neo* was used to load and manipulate the data in the analysis script. ***A***, Description of the experimental protocol (cf. Brochier et al., 2018 for details). The monkey is instructed to grasp an object using either a SG or PG, and this is identified as the CUE-ON event. After a 1 s delay, a GO signal (GO-ON event) marks the start of the movement and indicates whether to pull the object with either low (LF) or high (HF) force. Several behavioral events occur during a trial (marked on the time line). ***B***, Overview of the *Neo* data model defining container objects (shown here: *Block* and *Segment*) and data objects (shown here: *AnalogSignal* and *Event*). Supporting metadata are stored as *annotations* and *array_annotations* dictionaries (gray shading). Annotations are single values associated with a key (e.g., *subject_name*). Array annotations are stored in arrays with the length of the data (e.g., number of events in *Event* or number of channels in *AnalogSignal*). *Segment* objects group data objects in specific windows of time (given by attributes *t_start* and *t_stop*). *Event* objects are arrays with timestamps of labeled events. *AnalogSignal* objects are two-dimensional arrays (first dimension: time, second dimension: channels) where the time axis is determined by the *t_start* and *sampling_rate* attributes. The *units* attribute identifies the physical unit associated with the data array (e.g., microvolts for *AnalogSignal* and seconds for *Event*). ***C***, Time-homogeneous representation of two data elements of the dataset (*AnalogSignal* and *Event*) stored inside a *Segment* container. Selected timestamps and corresponding values of the array annotation *trial_event_labels* of the *Event* object are presented in blue. Image in panel A is adapted from Brochier et al. (2018), licensed under the Creative Commons Attribution 4.0 International License.

As a use case scenario, we consider an analysis that computed the mean power spectral densities (PSDs) from a publicly available dataset containing massively parallel electrophysiological recordings (raw electrode signals, local field potentials, and spiking activity) in the motor cortex of monkeys in a behavioral task involving movement planning and execution. The experiment details, data acquisition setup, and resulting datasets were previously described (Brochier et al., 2018). Briefly, two subjects (monkey N and monkey L) were implanted with one Utah electrode array (96 active electrodes) in the primary motor/premotor cortices. Subjects were trained in an instructed delayed reach-to-grasp task. In a trial, the monkey had to grasp a cubic object using either a side grip (SG) or a precision grip (PG). The SG consists of the subject grasping the object with the tip of the thumb and the lateral surface of the other fingers, on the lateral sides of the object. The PG consists of the subject placing the tips of the thumb and index finger on a groove on the upper and lower sides of the object. The monkey had to pull the object against a load that required either a low (LF) or high pulling force (HF). The grip and force instructions were presented through a light-emitting diode (LED) panel using two different visual cue signals (CUE and GO), respectively, which were separated by a 1,000 ms delay (Fig. 2*A*). As a result of the combination of the grip and force conditions, four trial types were possible: side grip with low force (SGLF), side grip with high force (SGHF), precision grip with low force (PGLF), and precision grip with high force (PGHF). A recording session consisted of several repetitions of each trial type that were acquired continuously in a single recording file. Neural activity was recorded during the session using a Blackrock Microsystems Cerebus data acquisition system, with the raw electrode signals bandpass-filtered between 0.3 and 7,500 Hz at the headstage level and digitized at 30 kHz with 16-bit resolution (0.25 V/bit, raw signal). The behavioral events were simultaneously acquired through the digital input port that stored 8-bit binary codes as received from the behavioral apparatus controller.

The experimental datasets are provided in the *Neuroscience Information Exchange* (*NIX*) format (RRID:SCR_016196; https://nixio.readthedocs.io), developed with the aim to provide standardized methods and models for storing neuroscience data together with their metadata (Stoewer et al., 2014). Inside the *NIX* file, data are represented according to the data model provided by the *Neo* (RRID:SCR_000634; https://neuralensemble.org/neo) *Python* library (Garcia et al., 2014). *Neo* provides several features to work with electrophysiology data. First, it allows loading data files written using open standards such as *NIX* as well as proprietary formats produced by specific recording systems (e.g., Blackrock Microsystems, Plexon, Neuralynx, among others). Second, it implements a data model to load and structure information generated by the electrophysiology experiment in a standardized representation. This includes time series of data acquired continuously in samples (such as the signals from electrodes or analog outputs of a behavioral apparatus) or timestamps (such as spikes in an electrode or digital events produced by a behavioral apparatus). Third, *Neo* provides typical manipulations and transformations of the data, such as downsampling the signal from electrodes or extracting parts of the data at specific recording intervals. The objects may store relevant metadata, such as names of signal sources, channel labels, or details on the experimental protocol. In this use case scenario, *Neo* was used to load the datasets and manipulate the data during the analysis.

The relevant parts of the structure and relationships between objects of the *Neo* data model are briefly represented in Figure 2*B*. The *Neo* library is based on two types of objects: data and containers. Different classes of data objects exist, depending on the specific information to be stored. Data objects are derived from *Quantity* arrays that are provided by the *Python* *quantities* package (https://github.com/python-quantities/python-quantities) and provide *NumPy* arrays with attached physical units. The *AnalogSignal* is used to store one or more continuous signals (i.e., time series) sampled at a fixed rate, such as the 30 kHz raw signal captured from each of the 96 electrodes in the Utah array. The *Event* object is used to store one or multiple labeled timestamps, such as the behavioral events throughout the trials acquired from the digital port of the recording system. The container objects are used to group data objects together, and these are accessed through specific collections (lists) present in the container. The top-level container is the *Block* object that stores general descriptions of the data and has one or more *Segment* objects accessible by the *segments* attribute. The *Segment* object groups data objects that share a common time axis (i.e., they start and end within the same recording time, defined by the *t_start* and *t_stop* attributes; Fig. 2*C*). The *Segment* object also has collections to store specific data objects: *analogsignals* is a list of the *AnalogSignal* data objects, and *events* is a list of the *Event* data objects.

The *Neo* data model also defines a framework for metadata description as key-value pairs for its data and container objects through annotations and array annotations. Annotations may be added to any *Neo* object. They contain information that are applicable to the complete object, such as the hardware filter settings that apply to all channels contained in an *AnalogSignal* object. Array annotations may be added to *Neo* data objects only. They contain information stored in arrays, whose length corresponds to the number of elements in the data. They are used to provide metadata for a particular element in the data stored in the object. For instance, in the *Event* object representing the behavioral events in the reach-to-grasp task, the *trial_event_labels* array annotation stores the decoded event string associated with each event timestamp stored in the object (Fig. 2*C*). In the end, all the data in the *NIX* dataset are loaded into *Neo* data objects that encapsulate all the relevant metadata.

**Figure 3.**
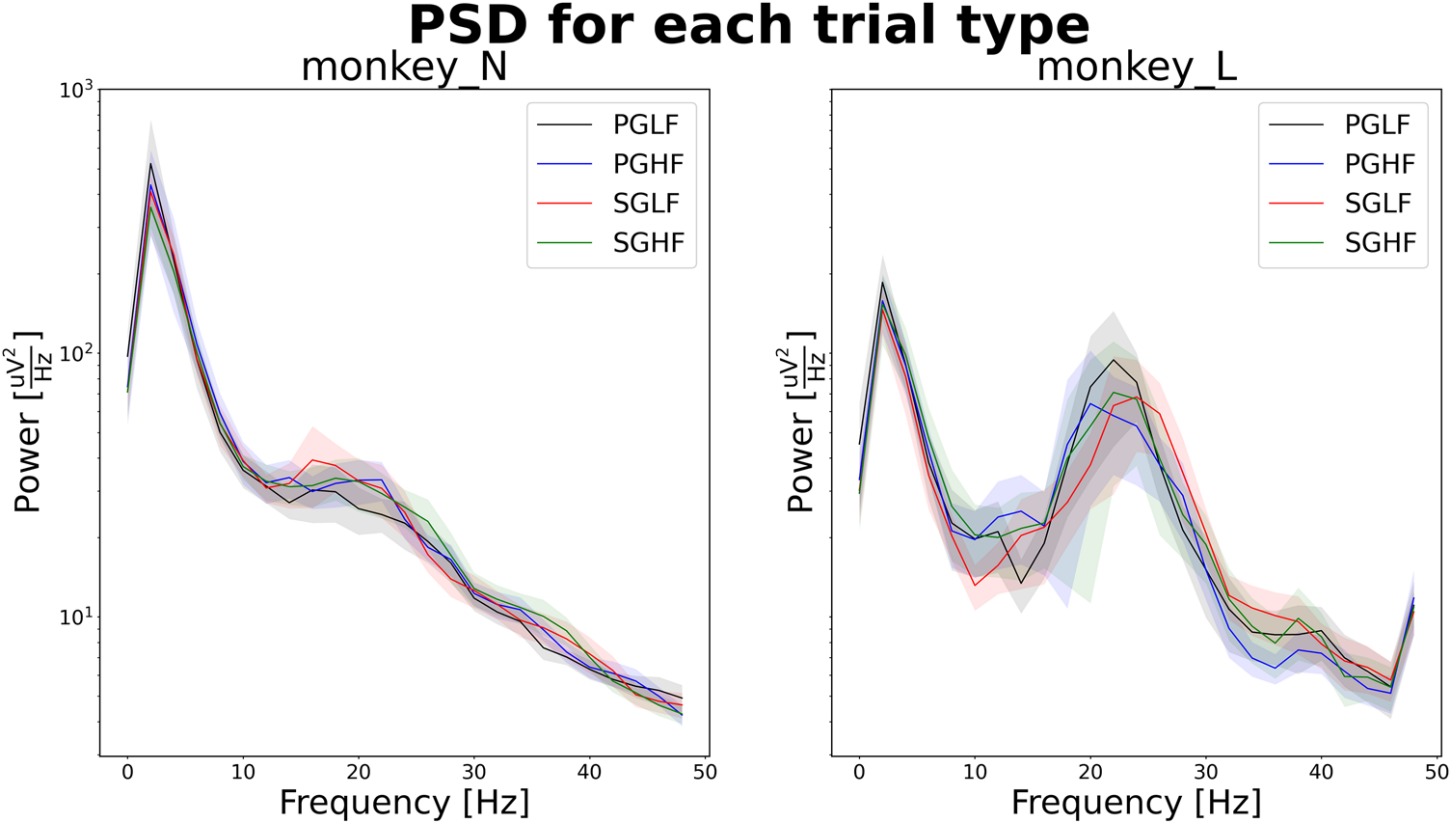
Output of the analysis workflow implemented in the script *psd_by_trial_type.py* in the context of the use case scenario. This plot was stored as a Portable Network Graphics (PNG) file named *R2G_PSD_all_subjects.png*.

**Figure 4.**
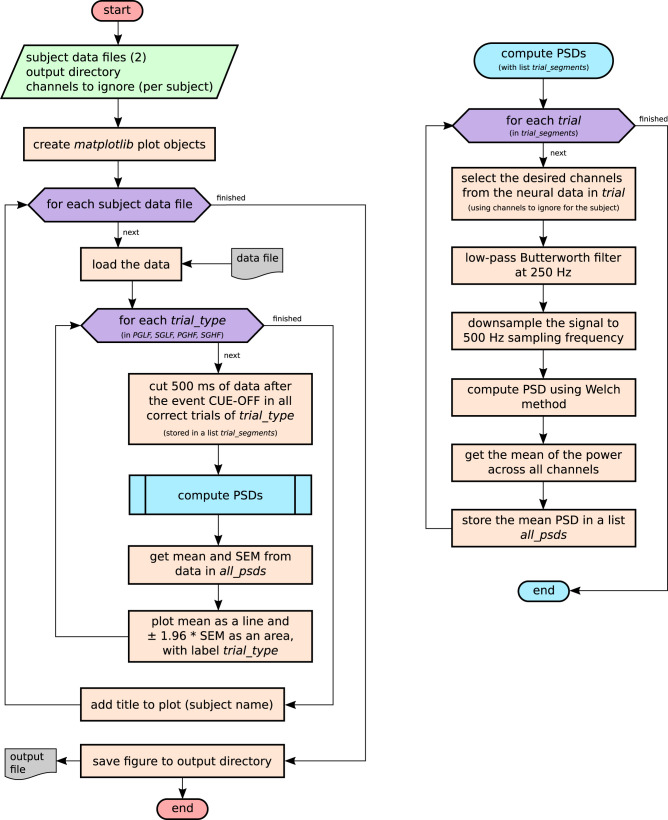
Flowchart of the analysis implemented as use case scenario. The code for this algorithm is implemented in *psd_by_trial_type.py*. The main steps are composed by three nested loops (purple hexagons): for each input file, a second loop runs over the four possible trial types, extracting the data of the individual trial epochs. After that, for each trial, the channel-wise power spectral densities (PSDs) are computed and the power density estimates from all channels are subsequently aggregated. At the end of each trial type loop, the single-trial PSDs are aggregated and plotted. After the last input file is processed, the plot is saved to *R2G_PSD_all_subjects.png*.

In the use case scenario, the PSDs were analyzed for each subject (monkey N and monkey L), and the mean PSD was computed for each of the four trial types present in the experiment (Fig. 3). Although a single *Python* script (named *psd_by_trial_type.py*) was used to produce the plot (stored as *R2G_PSD_all_subjects.png*), the actual analysis algorithm is complex (shown in a schematic form in Fig. 4). In a typical scenario, a file such as *R2G_PSD_all_subjects.png* could be stored in a shared folder or even sent to collaborators by e-mail. At this point, several key information cannot be obtained from the plot alone: (i) How were the trials defined, i.e., which time points or behavioral events were used as start and end points to cut the data in the data preprocessing? (ii) Was any filtering applied to the raw signal, before the computation of the PSD? (iii) Several methods are available to obtain the PSD estimate, each with particular features that may affect the estimation of the spectrum (Welch, 1967; Percival and Walden, 1993). Which method was used in this analysis, and what were the relevant parameters (e.g., for frequency resolution)? (iv) How was the aggregation performed (i.e., method and number of trials). What do the shaded area intervals around the plot lines represent? In addition to these questions, the contents of a plot such as *R2G_PSD_all_subjects.png* may be the result of several iterations of exploratory analyses and development of *psd_by_trial_type.py*. In our scenario, parameters that could have been iteratively probed or improved could be the identification of failed electrodes, definition of a suitable time window for cutting the data from a full trial, or to select specific filter cutoffs. Therefore, *R2G_PSD_all_subjects.png* could be overwritten after *psd_by_trial_type.py* was run with different parameters or different versions of the code. Altogether, the exhaustive set of steps and definitions used for the generation of the analysis result is not apparent from *R2G_PSD_all_subjects.png*. Even with a good description such as the flowchart in Figure 4, which could be added as accompanying documentation, the exact parameters used for function calls are still missing, especially if these were determined during run time (such as the number of trials in the dataset).

**Figure 5.**
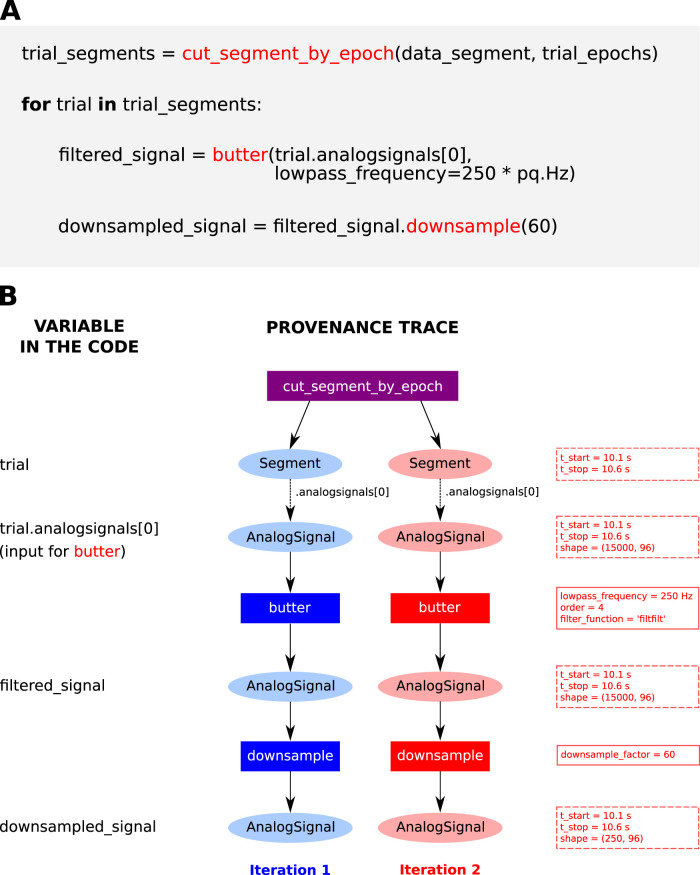
Provenance helps to understand exact data processing during code execution. ***A***, Snippet of code in *Python* that iterates over a list with individual trial data to perform a low-pass filter operation using a Butterworth filter followed by downsampling the signal by a factor of 60. In this example, the code uses data objects defined by the *Neo* framework (Garcia et al., 2014). The list *trial_segments* stores several *Segment* objects. The raw neural signal from the electrode array is stored in *AnalogSignal* objects. Note that although knowing the hierarchical structure of *Neo* helps to understand the implemented code, the actual data objects and any associated information cannot be accessed, as these exist only during run time. ***B***, Example of a provenance trace to represent the execution of the code in ***A***. Data objects are represented as ellipses, and functions as rectangles. Two exemplary iterations of the loop are shown as two separate paths in a graph (highlighted by blue or red color shades, respectively). Dashed lines represent accessing a data object contained into another data object using a specific *Python* operation (e.g., subscript or attribute). Note that the *Segment* object stored in the variable *trial* is known for every single iteration, and its transformations are followed individually. The information associated with each data object (e.g., start and end time of *Segment*, or the shapes of the *AnalogSignal* objects) can be inspected at every stage of the iteration (dashed red rectangles on the right show information for the second loop iteration). Parameters of the functions called, even if they were not explicitly written in the code, but determined during run time, can also be inspected (solid line red rectangles on the right show parameters for each function called in the second iteration).

The only way of getting those relevant details of the analysis is by directly inspecting *psd_by_trial_type.py*. The difficulties associated with this approach are illustrated in Figure 5. For a simple code snippet (Fig. 5*A*), which iterates over a list of trial data to apply a Butterworth filter and then downsample the signal, it is not possible to visualize the state of the data for each iteration (e.g., the array shape). In addition, the actual contents of the variables are unknown. A robust data model like *Neo* helps to understand which objects were accessed during each iteration. However, even when using that framework, the exact data objects and their transformations in each iteration of the for-loop are not apparent from the code given that the object instances (including attributes, such as the shape of an array) are only available during run time. One example of such information that exists only at run time is the number of trials (i.e., the number of *Segment* objects returned by *cut_segment_by_epoch*) and the number of channels (i.e., the shape of the *AnalogSignal* object in each loop iteration). Unless running the script again with the same dataset and explicitly outputting this information, it is not possible to know. In contrast, by capturing and structuring the relevant provenance during the execution, a representation could be obtained in a way that all relevant information is accessible after the run (Fig. 5*B*). The detailed trace ultimately shows which part of the data and the resulting intermediate objects were used during each iteration.

### Alpaca: a tool for automatic and lightweight provenance capture in *Python* scripts

As the analysis of electrophysiology datasets is usually based on scripts such as *psd_by_trial_type.py*, we set to implement Alpaca (Automated Lightweight ProvenAnce CApture) as a tool to capture the provenance information that describes the main steps implemented in scripts that process data. The captured information can be stored as a metadata file that is associated with the result file(s) generated by the script (e.g., the plot in Fig. 3 stored in *R2G_PSD_all_subjects.png*). Alpaca can be used for scripts written in the *Python* programming language as *Python* is free and open source, and has been gaining popularity among the neuroscience community (Muller et al., 2015). *Python* is also frequently used in the analysis of electrophysiology data, and several dedicated open source packages are available, such as the *Neo* and *NWB* (Neurodata without borders; RRID:SCR_015242; https://www.nwb.org) (Rübel et al., 2022) frameworks for electrophysiology data representation, the unified spike sorting pipeline *SpikeInterface* (RRID:SCR_021150; https://spikeinterface.readthedocs.io) (Buccino et al., 2020), and *Elephant* (Electrophysiology Analysis Toolkit; RRID:RRID:SCR_003833; https://python-elephant.org) (Denker et al., 2018) for data analysis. Therefore, a tool implemented in *Python* will have greater impact in the neuroscience community, as no licenses or fees are required and it builds on already established state-of-the-art processing and analysis tools.

**Figure 6.**
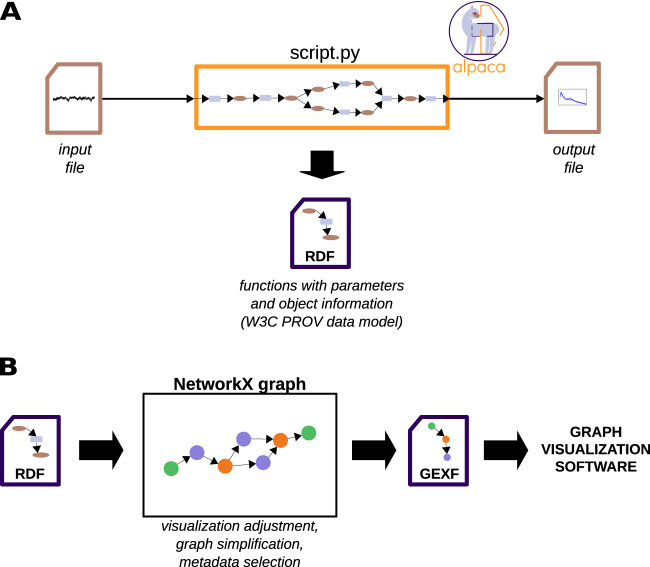
Schematic overview of the functionality of Alpaca. ***A***, The decorator and functions provided by Alpaca are incorporated into a *Python* script that processes data (orange rectangle). The script reads an input file and generates another file as result output. Alpaca tracks the functions called during the execution of the script (represented by the light blue rectangles inside the orange rectangle) together with the input and output data objects (represented by the brown ellipses within the orange rectangle). Function parameters and metadata of data objects are also captured. The aggregated information is structured according to the W3C PROV standard and serialized to a file (dark blue) using Resource description framework (RDF) acting as a sidecar file to the output file of the script. ***B***, To visualize the captured provenance, the serialized RDF files can be converted into *NetworkX* graph objects using Alpaca. The graph can be adjusted to concentrate on specific information of the captured provenance, or simplified. Finally, the graph can be saved using graph serialization formats (e.g., Graph exchange XML format; GEXF) supported by third-party graph visualization software (e.g., *Gephi*).

The functionality of Alpaca is illustrated in Figure 6. Alpaca is based on a *Python* function decorator (a *Python* decorator allows adding new functionality to existing functions without changing their behavior) that supports tracking the individual steps of the analysis and constructing a provenance trace. In addition, Alpaca serializes the captured provenance information (Fig. 6*A*) as a metadata file encoded in the RDF format (Resource Description Framework, a general model for description and exchange of graph data; Adida et al., 2015) according to the data model defined in the W3C (World Wide Web Consortium; https://www.w3.org) PROV standard (PROV-DM; Belhajjame et al., 2013). PROV is an open standard that was developed to allow the interoperability of provenance information in heterogeneous environments (Groth and Moreau, 2013). Finally, visualization of the provenance trace is supported by converting the PROV metadata into graphs that show the data flow within the script and allow the visual inspection of the captured provenance (Fig. 6*B*). Alpaca is provided as a standalone open source *Python* package that can be installed from the *Python Package Index* or directly from the code repository (https://github.com/INM-6/alpaca). The documentation with usage examples is available online (https://alpaca-prov.readthedocs.io).

Several design decisions were adopted in Alpaca. First, the tool captures provenance during the execution without the need for users to enhance this information with additional metadata or documentation. Second, code instrumentation is reduced to a minimum level, and users are asked to make only minor changes in the existing code to enable tracking (see the online document contained within the code repository accompanying this study (https://github.com/INM-6/alpaca_use_case/blob/f1696ec8dceaadbed6b825636ca7eb9aee704c92/documents/code_changes.pdf) showing the changes required to track provenance within *psd_by_trial_type.py*). Third, it is flexible enough to accommodate different coding styles, and it was designed to be the most compatible with existing code bases. Therefore, provenance is captured in an automatized and lightweight fashion.

Alpaca assumes that an analysis script such as *psd_by_trial_type.py* is composed of several functions that are called sequentially (potentially in the context of control flow statements such as loops), each performing a step in the analysis. The functions in the script may take data as input and produce outputs based on a transformation of that data, or generate new data. Moreover, a function may have one or more parameters that are not data inputs but modify the behavior on how the function is generating the output. For example, in reshaping an array using the *NumPy* function *reshape*, the new shape would represent a parameter that defines how to reshape the original array (i.e., input data) into a new array (i.e., the output data). In *Python*, information to a function is passed through function arguments that are accessed by the local code in the function body that performs the computation. Those are specified in the function declaration using the *def* keyword. Therefore, Alpaca utilizes the following definitions to analyze a function call in the script:

- *Input:* a file or *Python* object that provides data for the function. It is one of the function arguments;
- *Output:* a file or *Python* object generated by a function. Can be a return value of the function or one of the function arguments;
- *Parameter:* any other function argument that is neither an input nor an output;
- *Metadata:* additional information contained in the input/output. For *Python* objects, these can be accessible by attributes (i.e., accessed by the dot . after the object name, such as *signal.shape*) or annotations stored in dictionaries accessed by special attributes, such as the ones defined in the *Neo* data model. For files, this is the file path.

#### Initializing Alpaca

The calls to the functions tracked by Alpaca are expected to be present in a single scope (i.e., the main script body or a single function such as *main*). To identify the code to be tracked and start the capture, the user must insert a call to the **activate** function at a point in the script before the corresponding block of code. When calling **activate**, Alpaca identifies the current script in execution, obtains the SHA256 hash (a hash is a function that maps data with variable size to fixed-size values. SHA256 is a Secure Hash Algorithm (SHA) that can be used to verify the identity of files) of the source file storing the code, and generates a universally unique identifier (UUID) to identify the script execution (***session ID***). The source code to be tracked will be analyzed to allow the extraction of each individual code statement later, during the analysis of each function execution.

Before activating the tracking, the user can set options using the **alpaca_settings** function. These settings operate globally within the toolbox and control how Alpaca captures and describes provenance.

#### Tracking the steps of the analysis

**Figure 7.**
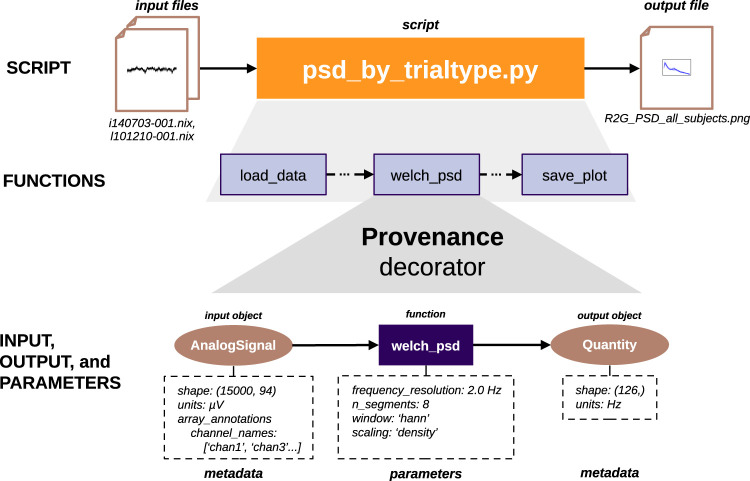
Alpaca captures fine-grained provenance information at each step during the execution of *Python* scripts that process data. The **Provenance** decorator is used to wrap each function called in the script. The input and output data objects for each function are identified, and any embedded object metadata is captured (bottom right and left). Object metadata are attributes of the objects or special values stored in annotation dictionaries. This takes advantage of available data models for electrophysiology where experimental details can be stored together with the data as annotations (e.g., *Neo*). In the example provided in this paper, the object metadata are attributes such as shape and units, together with annotations such as channel names in an *AnalogSignal* with the data recorded from the electrodes. Finally, the parameters of the functions are also identified (bottom middle). In this example, this is the type of window or the number of segments used in the computation of the PSD using the *welch_psd* function that implements the Welch algorithm. In the end, a full provenance graph with the lineage from the input files (such as the two Neuroscience Information Exchange (*NIX*) files in the example) to the output file with the analysis result (such as *R2G_PSD_all_subjects.png*) is produced.

The **Provenance** function decorator is used to wrap each data processing function executed in the script (Fig. 7). When applying the decorator, the argument names that are either *Python* object inputs, file inputs, or file outputs are identified through the decorator constructor parameters **inputs**, **file_input**, or **file_output**. When the script is run, for each execution of the function, the decorator: (i) generates a description of the inputs and outputs, (ii) records the parameters used in the call, (iii) generates a unique execution UUID (***execution ID***), and (iv) captures the start/end timestamps. Finally, this information is used to build a record for the function execution. **Provenance** has an internal global function execution counter, incremented after the execution of any function being tracked. The current value is also added to the function execution record to obtain the order of that execution. Finally, all the execution records are stored in an internal history, which will be used to serialize the information at the end.

The **Provenance** decorator analyzes the inputs and outputs to extract the information relevant for their description and their metadata:

- for *Python* objects (e.g., an *AnalogSignal* object), the type information (*Python* class name and the module where it is implemented), content hash, and current memory address are recorded. The content hash is computed using either the *hash* function from the *joblib* (https://joblib.readthedocs.io) package (using the SHA1 algorithm) or the builtin *Python* *hash* function (that uses the algorithm implemented in the *__hash__* method of the object). By default, every object will be hashed using *joblib*. However, it is possible to define specific packages whose objects will be hashed using the builtin *hash* function using the **alpaca_settings** function. This allows selecting hashing functionality that may already be implemented in the object (which can be faster), or avoid sensitivity to minor changes to the object content that will produce a provenance trace that is too detailed. The values of all object instance attributes (i.e., stored in the *__dict__* dictionary) are recorded, together with the values of the specific attributes when present. This includes, for example, *shape* and *dtype* for *NumPy* arrays, or extended attributes such as *units*, *t_start*, *t_stop*, *nix_name*, and *dimensionality* for the *AnalogSignal* object of *Neo* representing a measurement time series. More generic attributes that could be used by other data models, such as *id*, *pid*, or *create_time*, are also captured if present. Currently, the support to capture extended metadata details is implemented for *NumPy*-based objects;
- for files, the SHA256 file hash is computed using the *hashlib* package, and the absolute file path is recorded;
- for the *Python* builtin *None*, the object hash is an UUID, as it is a special case where the actual object is shared throughout the execution environment. This avoids duplication.

The information on the function is also extracted: name, module, and version of the package where it was implemented (if available through the *metadata* module from the *importlib* package implemented in *Python* 3.8 or higher). Version information is currently not recorded for user-defined functions (i.e., implemented in the script file being tracked).

Finally, the inputs to a function may be accessed from container objects by subscripts (e.g., an item in a list such as *signals[0]*) or attributes (e.g., *segment.analogsignals*). To capture these static relationships, the abstract syntax tree of the source code statement containing the current function call is analyzed, all container objects are identified, and the operations (subscript or attribute) are added to the execution history. In the end, the container memberships are identified and recorded if used when passing inputs to a function.

#### Serialization of the provenance information

The captured provenance is serialized as RDF graph (Adida et al., 2015), using one of the formats supported by *RDFLib* (https://github.com/RDFLib/rdflib). The **AlpacaProvDocument** class is responsible for managing the serialization, based on the history captured by the **Provenance** decorator. For simplified usage, the serialization can be accomplished in a single step by just calling the **save_provenance** function at the end of the script execution, passing a destination file and serialization format. All the information currently stored in the history in **Provenance** will be saved to the disk.

**Figure 8.**
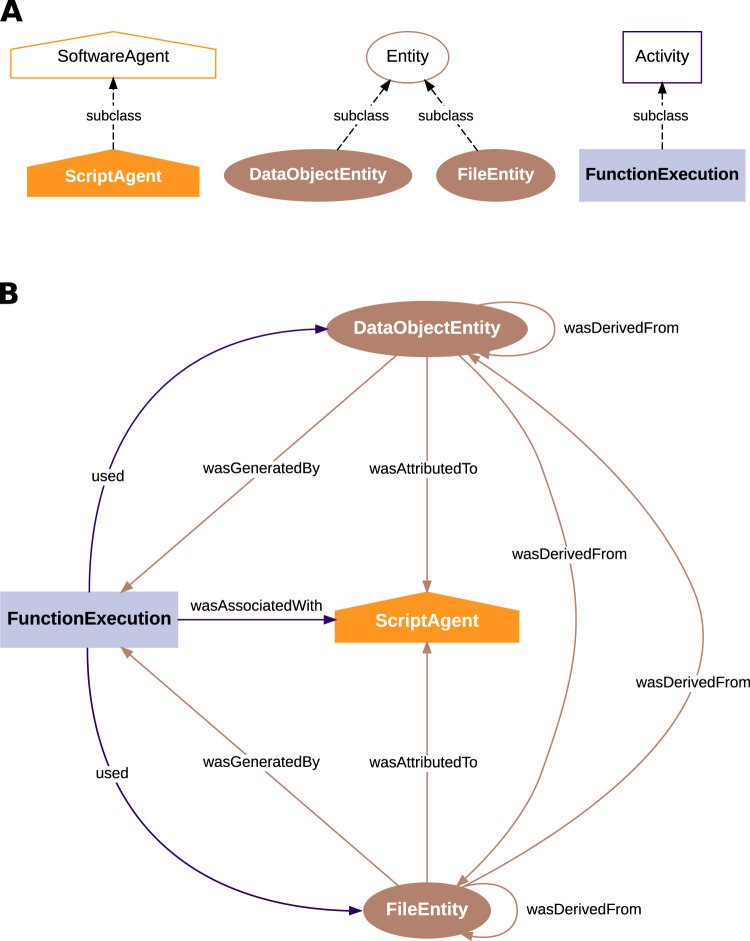
The Alpaca ontology used to serialize provenance information. ***A***, Main classes (bottom, filled shapes) derived from PROV-O (top, unfilled shapes). Objects storing data and files are represented as PROV-O Entities. The execution of a function is a PROV-O Activity. The script is a PROV-O SoftwareAgent (which in turn is derived from the PROV-O Agent class). ***B***, PROV-O provenance relationships among the classes in the Alpaca ontology.

For the RDF representation of the captured provenance, the PROV-O ontology (Lebo et al., 2013) was extended to incorporate properties relevant to the description of the provenance elements captured by Alpaca. Figure 8*A* shows the main classes derived from the SoftwareAgent (a subclass of Agent), Entity, and Activity classes of the PROV-O ontology, and Figure 8*B* shows the provenance relationships among the classes, as defined in PROV-O. These main classes are:

- **DataObjectEntity**: entity used to represent a *Python* object that was an input or output of a function;
- **FileEntity**: entity used to represent a file that was an input or output of a function;
- **FunctionExecution**: activity used to represent a single execution of one function with a set of parameters;
- **ScriptAgent**: agent used to represent the script that was run and executed several functions in sequence.

In addition to the classes derived from PROV-O, two additional classes are defined in the Alpaca ontology. They are used to represent specific information in the context of the provenance captured by Alpaca:

- **Function:** represents a *Python* function. It contains code that is executed to perform some action in the script, and that can take inputs, parameters, and produce outputs (e.g., in our example, the *welch_psd* function defined in the *spectral* module of the *Elephant* package);
- **NameValuePair:** represents information where a value is associated with a name. Name is a string and value can be any literal (e.g., integers, strings, decimal numbers). This is the main class used to store function parameters and data object metadata.

The Alpaca ontology also defines specific extended properties which are used to serialize function parameters, object/file metadata, and function information. They are summarized in Table 1.

**Table 1.** Properties of the classes defined by the Alpaca ontology

Class	Property	Description	Value
DataObjectEntity	hasAnnotation	The value of an annotation present in the object. Annotations are stored in dictionaries accessible by either the *annotations* or *array_annotations* object attributes. The annotation name is the dictionary key, and the annotation value is the corresponding value. The support for annotations is currently supported for *Neo*. However, the functionality for complex data types is implemented as a plugin system to support for data objects from additional frameworks, such as *NWB* or *Pandas*.	NameValuePair
	hasAttribute	The value of an attribute of the object (i.e., accessible by the dot such as *signal.shape*)	NameValuePair
	hashSource	One of the three methods used to obtain the object hash: *joblib_SHA1*, *Python_hash*, or *UUID*	xsd:string
FileEntity	filePath	The absolute path where the file is located in the system	xsd:string
FunctionExecution	hasParameter	A parameter passed to the function when called	NameValuePair
	executionOrder	Value of the global execution counter when the function was executed	xsd:int
	codeStatement	Statement in the source code that originated the call to the function	xsd:string
	usedFunction	Function that was called	Function
ScriptAgent	scriptPath	Absolute path to the file containing the source code of the script being executed	xsd:string
Function	functionVersion	Version of the package where the function is implemented. If function information is not available, it will be *NA*.	xsd:string
	functionName	The name of the function, as written in the *def* statement of the *Python* function definition	xsd:string
	implementedIn	The full path to the module where the function is implemented (example: for the *rand* function defined in the *random* module of the *NumPy* package, the value of the property will be *numpy.random*)	xsd:string
NameValuePair	pairName	Name that identifies the value	xsd:string
	pairValue	Value that is associated with the name	rdfs:Literal

The prefix *xsd:* identifies the namespace of the Extensible Markup Language (XML) Schema and *rdfs:* the namespace of the RDF Schema. Values without a namespace indicated by a prefix are classes defined in the Alpaca ontology.

For representing memberships, such as objects accessed from attributes (e.g., *segment.analogsignals*), indexes (e.g., *signals[0]*), or slices (e.g., *signals[1:5]*), the PROV-O *hasMember* property is used. The **DataObjectEntity** representing the container object will have a *hasMember* property whose value is the **DataObjectEntity** representing the element accessed. The element will have one of the following properties to describe the membership:

- **fromAttribute:** a string storing the name of the attribute used to access the object in the container (e.g., *analogsignals* in *segment.analogsignals*);
- **containerIndex:** a string storing the index used to access the object in the container (e.g., *0* in *signals[0]*). This is not necessarily a number, as *Python* uses string indexes when accessing elements in dictionaries;
- **containerSlice:** a string storing the slice used to access the object (e.g., *1:5* in *signals[1:5]*).

In the RDF graph, each data object, file, or function execution is identified by a uniform resource name (URN) identifier (Saint-Andre and Klensin, 2017). The functions and script are also represented by their own URNs. To compose a unique identifier, specific information captured during the script execution is used in the composition of the final URN string. The *authority* identifier element is a string that points to the institute or organization which has responsibility over the analysis. It can be set using the **alpaca_settings** function. The identifiers generated by Alpaca are summarized in Table 2.

**Table 2.** Composition of URN identifiers for each element described in the Alpaca provenance records

A.	
Alpaca ontology class	Identifier
DataObjectEntity	urn:[authority]:alpaca:object:Python:[class name]:[object hash]
FileEntity	urn:[authority]:alpaca:file:[hash type]:[file hash]
FunctionExecution	urn:[authority]:alpaca:function_execution:Python:[script file hash]:[session ID]:[function name]#[execution ID]
Function	urn:[authority]:alpaca:function:Python:[function name]
ScriptAgent	urn:[authority]:alpaca:script:Python:[script file name]:[script file hash]#[session ID]
B.	
Identifier element	Description
Authority	String defining the authority associated with the records
Class name	Name of the object class in *Python*, with full module path from the source package where it is implemented
Object hash	Content hash of the *Python* object
Hash type	Method to hash the file (currently only SHA256 is supported)
File hash	Hash value of the file
Script file hash	SHA256 hash of the *Python* file containing the script source code
Session ID	UUID generated when activating Alpaca tracking (***session ID***)
Function name	Name of the function, with full module path from the source package
Execution ID	UUID generated during the execution of the function (***execution ID***)
Script file name	Name of the file containing the source code

A, general schema for the composition of the identifier associated with each class in the ontology. B, details of identifier parts mentioned between brackets in A.

**Figure 9.**
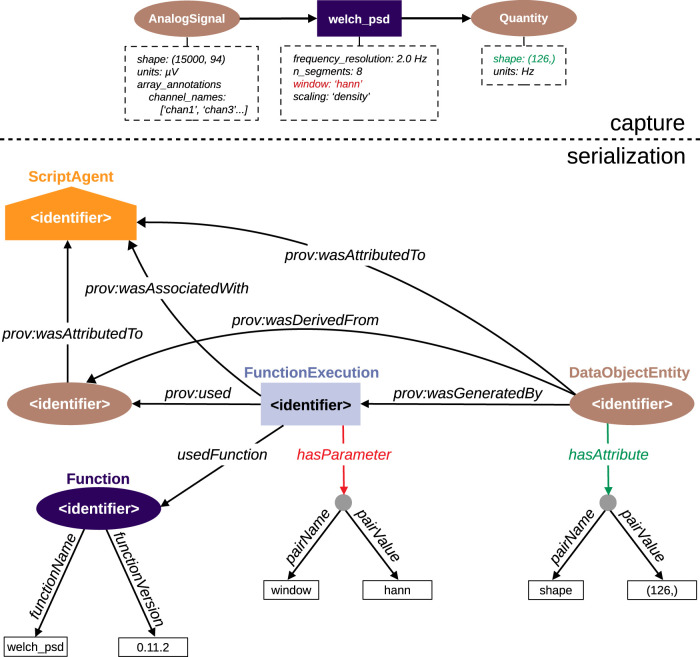
Example of the serialization of a single function execution with the Alpaca ontology. Top: information captured during the execution of a function (*welch_psd*) taking a *Neo* *AnalogSignal* as data input and returning a *Quantity* array. Bottom: the Alpaca ontology classes are used to represent the input/output objects and the function execution. The relationships from PROV-O are used to describe most relationships of the provenance. All elements are associated with the script as an agent. Object metadata and function parameters are serialized using the extended properties and relations provided by Alpaca (an example for a function parameter is shown in red using **hasParameter**, and for an object attribute in green using **hasAttribute**). In the diagram, the gray circles represent blank nodes of the **NameValuePair** class. Some additional properties captured and serialized during the function execution were omitted in the diagram here for clarity. *prov:* is the PROV-O namespace. Whenever a namespace is not defined, the class or property belongs to the Alpaca ontology.

Figure 9 summarizes how a single function execution is stored in the serialized RDF graph using the Alpaca ontology and the PROV-O properties.

#### Visualization of the serialized provenance

The provenance records serialized to RDF files can be loaded as *NetworkX* (RRID:SCR_016864; https://networkx.org) (Hagberg et al., 2008) graph objects. Besides the functionality for graph analysis offered by *NetworkX*, the graph objects can be saved as GEXF (Graph exchange XML format; https://gexf.net) or GraphML (http://graphml.graphdrawing.org) files that can be visualized by available graph visualization tools, e.g., *Gephi* (RRID:SCR_004293; https://gephi.org) (Bastian et al., 2009), or other *Python*-based frameworks, e.g., *Pyvis* (https://pyvis.readthedocs.io) (Perrone et al., 2020). This takes the advantage of existing free and open source solutions developed specifically for analyzing and interacting with graphs.

**Figure 10.**
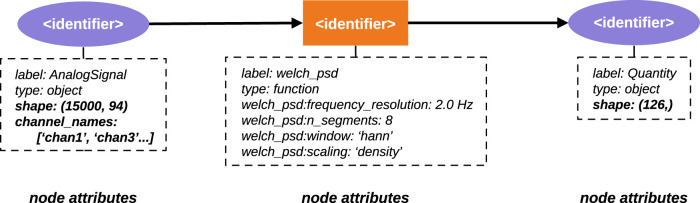
Example of a *NetworkX* graph generated from the RDF files serialized using the Alpaca ontology. This shows the expected nodes (two **DataObjectEntities** and one **FunctionExecution**) from the example in Figure 9. Node attributes of **DataObjectEntities** are included in the provenance trail if they are selected by the user when generating the graph using Alpaca functionality (attributes selected in the context of the use case scenario are shown in bold).

In Alpaca, the **ProvenanceGraph** class is responsible for generating the *NetworkX* graph objects from serialized provenance data. Figure 10 summarizes how the visualization graph is obtained from the RDF graph. The resulting graph will have entities (**DataObjectEntity** or **FileEntity**) and activities (**FunctionExecution**) as nodes, identified by the respective URN. Directed edges show the data flow across the functions. Metadata and function parameters are added to the attributes dictionary of each node. A few attributes are present for all the nodes in the graph (omitted in Fig. 10 for clarity):

- **type:** describes one of the three possible types of node: object, file, or function;
- **label:** for data objects, it is the *Python* class name (e.g., *AnalogSignal*). For functions, it is the function name (e.g., *welch_psd*). For files, it is *File*;
- **Python_name:** for data objects and functions, it is the full module path to the class or function, with respect to the package where it is implemented (e.g., *neo.core.analogsignal.AnalogSignal*). For files, this attribute is not used;
- **Time Interval:** a string representing a time interval according to the standard used by *Gephi* that is composed from the order of the function execution. This information can be used to visualize the temporal evolution of the provenance graph, e.g., using the timeline feature of *Gephi* that displays only the nodes within a specified execution interval.

The **ProvenanceGraph** class provides options to tweak the visualization. First, it is possible to select which attributes and annotations from the metadata to include in the visualization graph. Second, parameter names can be prefixed by the function name, so that they can easily be identified. Third, nodes representing the builtin *Python* *None* object (that is the default return value of a *Python* function) can be omitted. Finally, nodes describing a sequence of object access operations from containers (e.g., *segment.analogsignals[0]*, which accesses the list in the *analogsignals* attribute of *segment*, followed by retrieving its first element) can be condensed such that a single edge describing the operation is generated. These visualization options reduce clutter and facilitate the visual inspection of the recorded provenance information.

Finally, the provenance graphs can become large when repeated operations are performed within the script, such as using a *for* loop to iterate over several data objects to perform computations. Therefore, an aggregation and summarization are available, adapted from the functionality already implemented in *NetworkX* (from version 2.6). It uses the Summarization by Grouping Nodes on Attributes and Pairwise edges (SNAP) aggregation algorithm, and was modified from the original implementation to allow the selection of specific attributes of a set of nodes. Moreover, for functions executed with distinct sets of parameters, the different values can also be taken into account when identifying similarity of nodes in summarizing the graph. The aggregation generates supernodes that represent not a single execution and data, but several identical or similar processing nodes. The identifiers of the individual elements that were aggregated in the supernode are listed in the *members* node attribute. The total number of nodes aggregated into the supernode is stored in the *member_count* node attribute. In the end, the user can aggregate several nodes together, depending on whether they share the values of a given attribute, which allows the generation of a simplified version of the provenance trace that provides a more general overview of the analysis.

### Code accessibility

**Figure 11.**
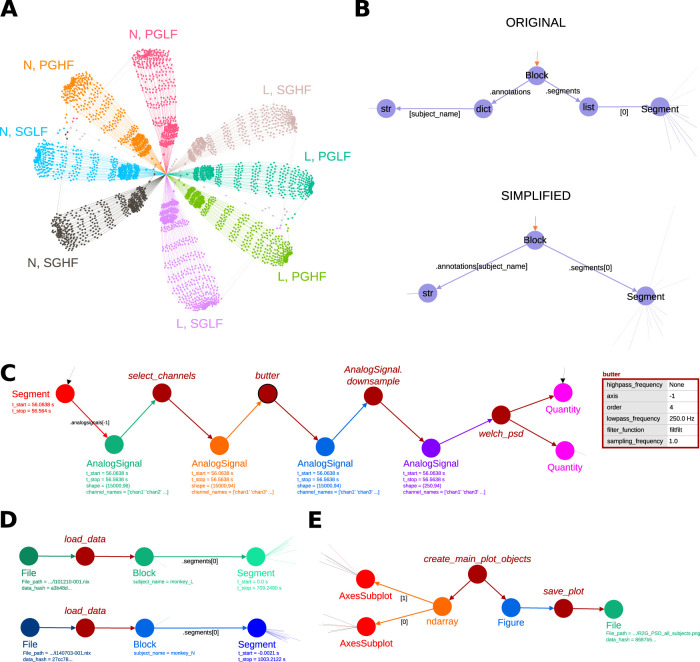
Overview of the provenance captured by Alpaca stored in *R2G_PSD_all_subjects.ttl*. ***A***, Visualization of the full (non-aggregated) graph corresponding to *R2G_PSD_all_subjects.ttl*. Each graph region (color coded) corresponds to the processing for a single subject (monkey N or monkey L) and trial type (PGHF, PGLF, SGHF, or SGLF). ***B***, Sequential object access operations are simplified such that only a single edge represents attribute and subscript access. Original example (top): addressing the first *Segment* object within the *Block* requires accessing its *segments* attribute, followed by retrieving the first element (index 0). Simplified version (bottom): the edge shows the operation as *.segments[0]*. The same simplification is applied to values stored in the annotations dictionary. ***C***, Processing of an individual trial. Details of data objects and function parameters can be inspected. Values of selected attributes and annotations are shown below the data node labels. Function nodes are dark red with labels in italic. Exact parameters for the *butter* function execution are shown in the table. ***D***, Loading of the *NIX* data file of each subject (monkey L: green; monkey N: blue). Function *load_data* (dark red) loaded each file and returned a *Block* object (*subject_name* annotation identifies the corresponding subject). The first *Segment* of the *Block* was used for further processing. ***E***, Generation of *R2G_PSD_all_subjects.png* from the *matplotlib* *Figure* object. In ***D*** and ***E***, the path and hash of files can be inspected.

**Figure 12.**
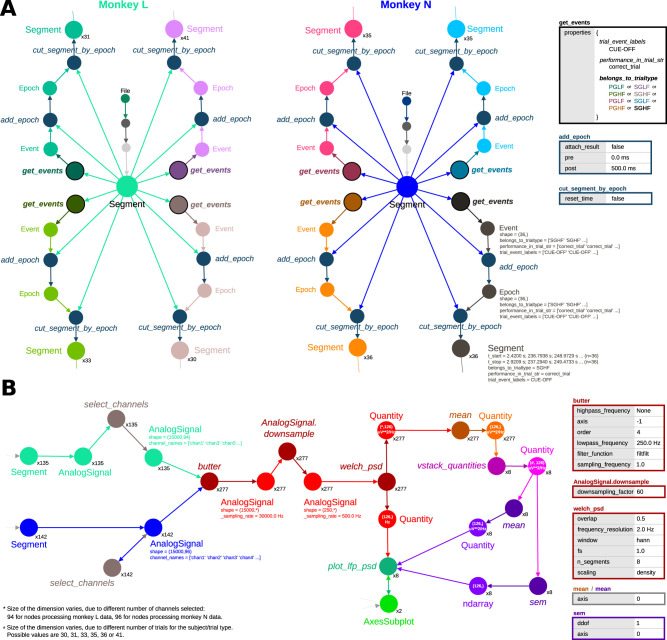
Alpaca captured the detailed provenance of *R2G_PSD_all_subjects.png*. Function nodes are labeled in italic and data objects in normal style. The multipliers next to a node show how many similar nodes were aggregated at that step. Selected function parameters are shown in the tables. ***A***, The aggregated graph demonstrates common steps in preprocessing the *Segment* objects containing data of monkey L (left) and monkey N (right). For each trial type, the *Neo* function *get_events* extracted the times of the CUE-OFF events of correct trials (see *properties* parameter in the table; dictionary key names are in italic). Each execution of *get_events* used distinct *belongs_to_trialtype* values (color in table matches color in graph). *Neo* functions *add_epoch* and *cut_segment_by_epoch* were called with same parameters across monkeys/trial types to extract data as *Segment* objects corresponding to 500 ms after CUE-OFF (dark blue). The number of trials can be inferred from the multipliers of the *Segment* objects. The details for the preprocessing of monkey N/SGHF trials are shown (dark gray). ***B***, Analysis steps after cutting data into trials (aggregated for each monkey/trial type). The values of the *shape* and *units* attributes of *Quantity* objects are shown inside the node. Function *select_channels* was executed taking the *AnalogSignal* with the electrode data for the trial (dark brown; *n* = 135 trials for monkey L and *n* = 142 trials for monkey N). For monkey L, channels 2 and 4 (*‘chan 2’* and *‘chan 4’* values in the *channel_names* array annotation) were removed. All trials (*n* = 277) were processed similarly (red shades), consisting of applying a 250 Hz low-pass Butterworth filter (function *butter*), downsampling to 500 Hz, and computing a PSD using the Welch method using a Hanning window with 50% overlap and 2 Hz frequency resolution (function *welch_psd*). For each *Quantity* array with power spectra, an average across channels was obtained (orange shades) and for each monkey/trial type (*n* = 8), those estimates were combined to obtain an array for that trial type (pink shades; the shapes of the arrays match the number of trials obtained in the preprocessing). Mean and standard error of the mean (SEM) across trials were obtained from these arrays (purple shades) and plotted (green shades).

**Figure 13.**
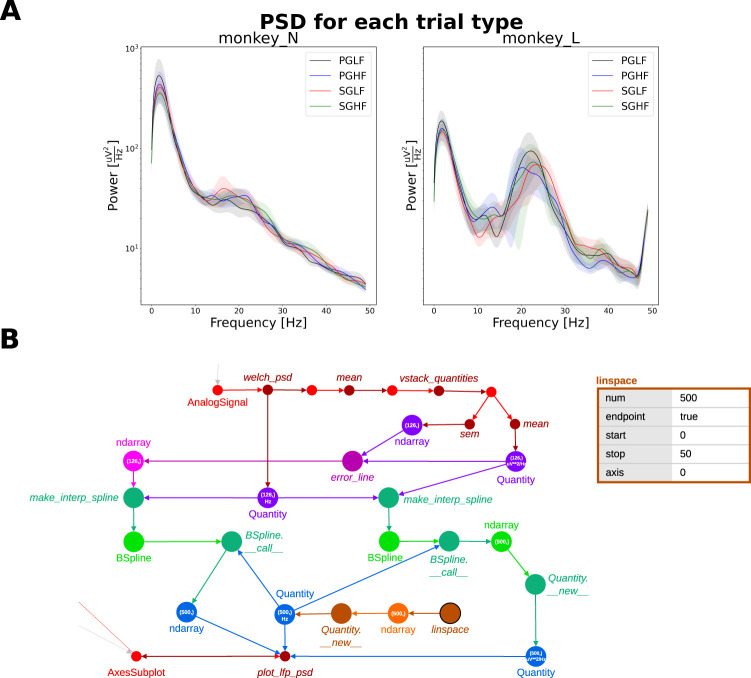
Provenance captured by Alpaca shows transformations of the results before plotting. ***A***, Alternate version of *R2G_PSD_all_subjects.png*. The lines appear smoothed in comparison to the plot shown in Figure 3. ***B***, Provenance track showing the steps after the computation and aggregation of the PSDs across channels and trials, before plotting with *plot_lfp_psd*. The visualization graph was aggregated to demonstrate the common steps when plotting the different estimates for each subject and trial type. Nodes in darker shades with labels in italic are function calls and nodes in lighter shades with labels in normal style are data objects. The steps also present in the generation of the plot in Figure 3 are highlighted in red (top; some labels omitted for clarity). The *NumPy* array (with SEM estimates) and *Quantity* arrays (with PSDs or frequencies) used in the original *R2G_PSD_all_subjects.png* are shown in purple (array shape and physical quantity are shown inside the nodes). In contrast to the generation of the original plot, an additional function computed the error line values (pink shades). Next, the *NumPy* arrays with error estimates or the *Quantity* arrays with PSD estimates were passed together with the *Quantity* array with frequencies to the *SciPy* function *make_interp_spline*, which generates a *BSpline* interpolation object (green shades show the interpolation steps). For the interpolation using *BSpline*, an array with 500 elements between 0 and 50 was generated with *NumPy* *linspace* function (orange shades; the function node is highlighted with a black border). The detailed parameters for *linspace* recorded by Alpaca are shown in the table. This *NumPy* array was converted to a *Quantity* array that was used with the interpolation objects to obtain the final error and PSD arrays used in the plots (blue nodes).

**Figure 14.**
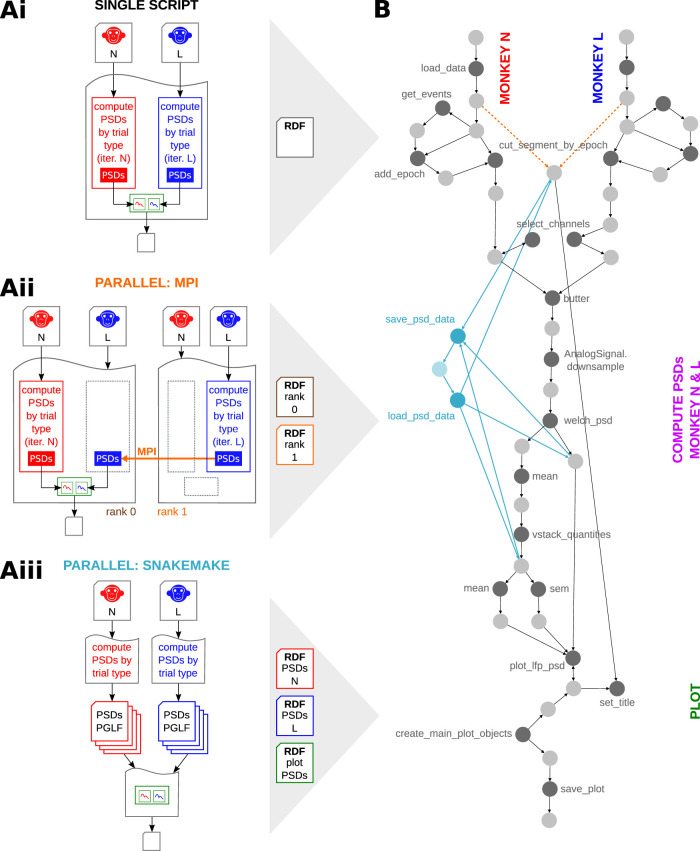
Alpaca can capture detailed provenance in parallelization and multi-script scenarios. ***A***, Schematic representation of three possible scenarios for the analysis presented as use case. ***Ai***, Single script, corresponding to the original version of *psd_by_trial_type.py*. The script is run with the two data files (N and L) as arguments. Each file is processed in a loop (red and blue rectangles), which computes the PSDs for each trial type (filled rectangles). The PSDs in memory are aggregated, plotted and saved as *R2G_PSD_all_subjects.png* (green rectangles). ***Aii***, *psd_by_trial_type.py* is modified to perform the analysis using message passing interface (MPI) parallelization. The script is run as two parallel processes, taking both data files as arguments. The main process (rank 0) performs operations involved in plotting the data from both subjects and saving *R2G_PSD_all_subjects.png* (green shapes). It executes only the loop iteration for monkey N (red rectangle). The second process (rank 1) executes only the loop iteration for monkey L (blue rectangle). At the end of the loop for rank 1, the PSD data in memory (filled blue rectangle) is transferred to the main process using MPI (orange arrow) to be aggregated and plotted. Alpaca generates 2 separate RDF files with the provenance of the execution of rank 0 and 1, respectively. ***Aiii***, *psd_by_trial_type.py* is broken into a multi-script workflow executed using *Snakemake*. The operations were split into 2 steps, each implemented as a single script. The first step reads a single data file, computes the PSDs for each trial type, and saves the in-memory data to files. This step corresponds to a single loop iteration in panel ***Ai*** (red and blue rectangles). The second step reads all the files, plots the PSDs, and saves *R2G_PSD_all_subjects.png* (green shapes). The execution produces 3 RDF files with provenance information. ***B***, The 2 RDF files of scenario ***Aii*** and the 3 files of scenario ***Aiii*** were trivially combined (concatenated) to obtain a single provenance graph for each scenario. For visualization, each provenance graph was automatically aggregated by Alpaca to show the main steps of the workflow. The aggregated graphs of scenarios ***Ai***–***Aiii*** are compared for the presence or absence of specific nodes and edges. Nodes in dark shades are function calls, and nodes in light shades are data objects (only labels of function names are shown for clarity). Nodes and edges that overlap in all three scenarios are shown in gray shades. The captured provenance shows a nearly identical structure in the three scenarios, with a fully connected graph describing the whole analysis process including data loading, data analysis, plotting and saving. In the MPI scenario, only an operation describing how the string to define the plot title was fetched from the *Block* annotations is missing (dotted orange edges). In the *Snakemake* scenario, few additional steps exist to save/load the data to/from the intermediate files (light blue nodes and edges).

The code to reproduce the analyses presented as use case in this paper is freely available online at https://github.com/INM-6/alpaca_use_case. Figures 1, 2, 4–10, and 14*A* were manually created using Inkscape. Figures 3 and 13*A* are direct outputs of the corresponding scripts. Figures 11, 12, 13*B*, and 14*B* were created from graph visualization files generated by the corresponding scripts (GEXF format). The GEXF files were loaded into Gephi (version 0.9.7) and nodes were edited for color, position, and size. The graphs were exported to Scalable Vector Graphics (SVG) files that were manually edited using Inkscape to compose the final figures. Editing involved adjusting label sizes and adding information available as node attributes in Gephi. The data used for the analysis can be found at https://gin.g-node.org/INT/multielectrode_grasp. All codes used in this manuscript are also available as Extended Data.

10.1523/ENEURO.0476-23.2024.d1 Extended Data 1 Download Extended Data 1, ZIP file.

10.1523/ENEURO.0476-23.2024.d2 Extended Data 2 Download Extended Data 2, ZIP file.

## Results

In the following, we will describe and evaluate the analysis provenance captured by Alpaca in the use case scenario described in Section 2.2. After running *psd_by_trial_type.py* with the code modified to use Alpaca, a detailed provenance trace was obtained and stored as *R2G_PSD_all_subjects.ttl*. Corresponding GEXF graph files for visualization were generated, with distinct levels of aggregation and granularity of the steps in *psd_by_trial_type.py*, ranging from a fine-grained view to a summarizing birds-eye view. The interactive analysis of those graphs using *Gephi* are presented in the form of a video (accessible at https://purl.org/alpaca/video). Here, we will present the main features of the provenance trace using several *Gephi* graph exports. Then, we detail how they address the four challenges for tracking provenance of the analysis we identified in the Materials and Methods and Figure 1.

### Overview of the captured provenance

Figure 11*A* shows the overview of the graph generated from *R2G_PSD_all_subjects.ttl* (*None* objects returned by functions were removed). Overall, 3,579 nodes and 4,313 edges are present, and the graph has eight colored regions. Each region corresponds to the iterations of the two outer loops in *psd_by_trial_type.py* (i.e., loop over two subjects × loop over four trial types resulting in eight iterations; Fig. 4). For the remainder of this study, the visualization is optimized to remove memberships due to the access of *Neo* objects in containers that introduces extra nodes in the graph. This simplification is illustrated in Figure 11*B*.

Using the timeline feature of *Gephi*, it is possible to isolate specific parts of the graph (Fig. 11*A*) based on the execution order of statements in the *Python* code. Here, we single out the time window that corresponds to the processing of a single trial in a loop iteration (Fig. 11*C*) and then inspect individual attributes of the objects and parameters of the functions involved until the computation of the PSD. It is possible to inspect the start and end time points of the trial segment with respect to the recording time in the dataset using the *t_start* and *t_stop* attributes of the *Segment* object at the beginning of the trace, thus uniquely identifying the analyzed data segment. It is also possible to review the *AnalogSignal* object containing the data that were later processed and used to compute the PSD by the *welch_psd* function of *Elephant*. General attributes, such as the shape of the data array of the *AnalogSignal* object, can be accessed together with specific metadata, such as the names of the channels associated with the time series in the data. Finally, for these intermediate steps, it is possible to inspect specific parameters passed to each function: the attributes of **FunctionExecution** graph nodes (shown example: *butter*) corresponding to function parameters are prefixed by the function name, followed by the name of the argument as defined in the *Python* function definition (cf., Fig. 10). Taken together, Alpaca captured these types of information for each individual step throughout the execution of *psd_by_trial_type.py* such that each iteration of the central analysis can be traced in detail after completion of the script.

It is possible to retrace the first steps after loading the two data files (Fig. 11*D*). A function called *load_data* (defined in *psd_by_trial_type.py*) was called with the neural data file (available as a dataset in the NIX file format) of one particular subject as input and returning a *Block* object with all the data of that recording session. We can inspect the *subject_name* annotation of *Block* and identify in human-readable text which subject corresponds to each object. We can alternatively bind each *Block* to the specific source data file, by inspecting the *File* node associated with each object, and obtain the SHA256 hash (*data_hash* node attribute). Although the actual path used in the analysis (*File_path* node attribute) will point to the actual location of the file in the system where the script was run, the hash will allow the identification of the file regardless of its name and location. Moreover, the graph shows that the first *Segment* stored in the *Block* was accessed (through the *segments* attribute of *Block*), and this was the main source for all subsequent analysis done for each monkey. By inspecting the node of the *Segment* object, we have access to its attributes and annotations, such as the starting and end times of the data in recording time (−0.0021 and 1003.2122 s for subject monkey N, and 0.0 and 709.2480 s for monkey L; Fig. 11*D*).

In a similar way as described above for reading the input data, we can inspect the generation of the output file (Fig. 11*E*). It was obtained from a *matplotlib* *Figure* object that was initialized by a function at the beginning of the script execution (*create_main_plot_objects*), was successively filled with graphs as power spectra were calculated, and finally saved to disk as a PNG file using a function called *save_plot*.

### Understanding the data preprocessing

Figure 12*A* shows the sequence of steps applied to the *Segment* object that contains the full data for one subject. When aggregated by function parameters (i.e., simplified based on similarity of function parameters), the graph shows four separate paths that start from each of the two *Segment* objects (one per subject). Each is comprised of the *Neo* functions *get_event*, *add_epoch*, and *cut_segment_by_epoch*. Each of those functions performs a specific action: identify specific events during the recording (stored in an *Event* object) according to selection criteria, select a window of data around these identified event timestamps (stored in an *Epoch* object), and finally use the windows stored in the *Epoch* object to cut the large *Segment*, producing one *Segment* object per epoch containing a window.

We can now analyze the captured provenance to verify the detailed parameters used in each of those preprocessing functions. *get_event* used a parameter called *properties*, together with the *Segment* object as input. That parameter defines a dictionary with keys and values that are compared to the annotations or attributes of a *Neo* *Event* object in order to select the desired subset of all events recorded during the experiment. All four paths considered the CUE-OFF event of correct trials (defined by the *trial_event_labels* = *‘CUE-OFF’* and *performance_in_trial_str* = *‘correct_trial’* dictionary entries). However, in each path, the function was called with the *belongs_to_trialtype* value containing one of the four possible trial labels: PGHF, PGLF, SGHF, or SGLF. Therefore, each *Event* object returned by *get_event* will contain the times of CUE-OFF of all correct trials of one of the four trial types.

The times of the generated *Event* objects were used to define epochs and cut the data to obtain segments of the trials of a particular type. Inspecting the subsequent executions of the functions *add_epoch* and *cut_segment_by_epoch*, their parameters show that epochs were defined as 500 ms after the CUE-OFF event (*pre* = *0.0* ms and *post* = *500.0* ms), and the absolute recording times were preserved when cutting (*reset_time=False*). Therefore, for each subject, we can partition the provenance graph in four separate paths, each dealing with processing data of a particular trial type (the outer loops of *psd_by_trial_type.py*; Fig. 2). Not only these selection criteria for extracting the data are retained by the provenance trail, but also we can retrieve the precise time points used for cutting the data and calculated only during run time based on the loaded data on a trial-by-trial basis (by inspecting the *t_start* and *t_stop* attributes of each *Segment* generated by *cut_segment_by_epoch*). Overall, Alpaca allowed us to understand the initial data preprocessing and trial definitions, addressing challenges 1 and 2.

### Inspecting the data flow used to generate a result

The figure stored in *R2G_PSD_all_subjects.png* and shown in Figure 3 could have been produced by different versions of *psd_by_trial_type.py*, with steps in different order or new steps added. A likely scenario is the necessity to filter out some channels for one of the datasets. In Figure 12*B*, we see that for each subject, a user-defined function called *select_channels* was applied to the data. For monkey L, it is apparent from the shapes of the data arrays that two recording channels were excluded (due to signal quality), such that only 94 of the 96 recording channels were used. The provenance track captured by Alpaca shows this, as the returned *AnalogSignal* object is different from the object containing all the channels, and the *shape* attribute shows the removal of the two channels.

At this point, it is possible to bind *R2G_PSD_all_subjects.ttl* to *R2G_PSD_all_subjects.png* through the SHA256 hash of the file written by the function *save_plot* (Fig. 11*E*). *R2G_PSD_all_subjects.ttl* will also have all the function executions linked to the script identifier, obtained from the hash of *psd_by_trial_type.py* and ***session ID*** (cf. Table 2). Thus, it was possible to record all operations within a single script together with the actual parameters used. In this way, the provenance information can be used to automatically capture and retain the ongoing development process from the perspective of the generated results, addressing challenges 2 and 3.

### Reviewing analysis parameters

In between runs of a single version of *psd_by_trial_type.py*, the analysis parameters could also have been changed, leading to alternate versions of the PSD estimates in *R2G_PSD_all_subjects.png* generated by each run. A scenario where this is likely to occur is one where the scientist performing the analysis may have iterated the code execution several times to find a set of parameters that allowed a good visualization of the power spectra.

The provenance track captured by Alpaca allows to inspect the values of each individual function call. From the *AnalogSignal* object after channel selection, there is a common pathway in the aggregated graph for both subjects (Fig. 12*B*). The functions *butter*, *AnalogSignal.downsample*, and finally *welch_psd* were called sequentially. Those correspond to the filtering, downsampling, and computation of the PSD using the Welch method. Each of those functions have key parameters that will affect the PSD estimate, and the parameters were captured automatically.

We can use the provenance information to verify that a 250 Hz low-pass cutoff was used for the filtering (from the parameter passed to the *Elephant* *butter* function). Moreover, we verify that the signal was downsampled by a factor of 60 (method *downsample* from the *AnalogSignal* object). By inspecting the shapes of the *AnalogSignal* objects that are input and output of the function, we can verify the downsample operation: the input object had 15,000 samples and, after *AnalogSignal.downsample*, the number was reduced to 250.

Finally, it is possible to inspect all the parameters for the PSD computation using the *Elephant* *welch_psd* function: a Hanning window was used, for an estimate with a 2 Hz frequency resolution. The resulting objects storing the frequency bins and power estimates (*Quantity* arrays) are discernible by the *units* attribute. The frequency array has a dimension of 126, which is expected for a PSD of a continuous signal downsampled to 500 Hz and with a frequency resolution of 2 Hz. It is also possible to observe that the power estimates are a two-dimensional array with first dimensions of 96 (for monkey N) and 94 (for monkey L), which agree with the source *AnalogSignal* objects and indicate the number of channels. Therefore, the power estimates were obtained for each channel as a single array.

Addressing challenges 1 and 2, it possible to retrieve the value of any parameter that may have resulted from trial and error iterations during the development of *psd_by_trial_type.py*, as the provenance information shows the detailed history of the generation of the data objects that were ultimately used by the plotting function.

### Facilitating sharing of analysis results

When sharing *R2G_PSD_all_subjects.png* with others, some parts of the figure leave guesswork to the collaborator. However, *R2G_PSD_all_subjects.ttl* contains several missing pieces of information that are not accessible from the figure stored in *R2G_PSD_all_subjects.png* alone.

In addition to the details of the analysis steps presented above, it is also possible to know the last steps used to transform the data before plotting the lines and intervals using the *plot_lfp_psd* function (Fig. 12*B*). First, an average of the power across all channels was obtained for each trial. The *NumPy* *mean* function was applied to the array with the per-channel power estimates, over the first axis (*axis* = *0* parameter). Then, the channel averages of all trials of the same trial type of a single subject were averaged in a grand mean (using the *NumPy* *mean* function). The individual trial averages were also used to obtain a SEM estimate (using the *SciPy* *sem* function). Finally, the grand mean and SEM were passed to the *plot_lfp_psd* function that performed the plotting in the *AxesSubplot* object corresponding to the graph panel for that subject, taking the multiplier 1.96 as a parameter to define the width of the intervals. Not only all these steps are now apparent, but it is also possible to know how many trials were used for each subject when plotting (monkey N: PGHF = 36, PGLF = 35, SGHF = 36, or SGLF = 35; monkey L: PGHF = 33, PGLF = 31, SGHF = 30, or SGLF = 41; Fig. 12*A* and *B*). In addition, for each call of *plot_lfp_psd* it is possible to inspect the parameter providing the legend label with respect to the source of the mean, SEM, and frequency data used as inputs.

As mentioned above, two electrode channels were excluded in the analysis of monkey L data. The provenance information in *R2G_PSD_all_subjects.ttl* makes it possible to check the *channel_names* annotations of each *AnalogSignal* object used in each iteration when computing the PSD (Fig. 12*B*). The inspected labels show that channels 2 and 4 were excluded for this monkey.

An additional scenario to illustrate how to make use of the captured provenance in a shared environment is presented in Figure 13*A*. Here, a plot resembling the one presented in Figure 3 is stored in *R2G_PSD_all_subjects.png*. However, the lines and interval area boundaries appear smoothed, suggesting the plot was generated by an alternate version of *psd_by_trial_type.py*. The provenance captured by Alpaca reveals steps after the aggregation of the power estimates across trials. Spline smoothing objects from the *SciPy* package were used to generate new arrays that were the inputs to the plotting function *plot_lfp_psd* (Fig. 13*B*). With this information, collaborators receiving *R2G_PSD_all_subjects.png* can clearly identify that the plot is not showing the actual estimates but a smoothed version.

In summary, addressing challenge 4, the provenance information captured by Alpaca facilitates sharing *R2G_PSD_all_subjects.png* as it provides additional information for finding and understanding the results without requiring extra work by the scientist performing the analysis.

### Provenance capture in parallelization and multiple-script scenarios

The complexity of electrophysiology analysis workflows can increase in multiple ways to accommodate the demands on data size and computational load of a particular analysis. In the following we explore in how far Alpaca can be integrated in two such scenarios, illustrated by the use case example (Fig. 14*Ai*).

One way is to adopt parallelization approaches inside a single script, such as using the message passing interface (MPI). In this approach, a script is run multiple times as separate simultaneous processes, and each run is given an identifier (rank). A script such as *psd_by_trial_type.py* could have the control flow modified, for example, such that each iteration in the main *for* loop processing the two subject files (i.e., monkey N or monkey L; Fig. 4) would be executed in different processes according to the rank value defined for that script execution (Fig. 14*Aii*). At the end of the loop iteration, the in-memory arrays with the computed PSDs are transferred to the main process (rank 0) using MPI routines to produce the plots and the final PNG file *R2G_PSD_all_subjects.png*. This approach allows the distribution of the execution of each iteration among the different compute cores.

A second way consists of breaking a complex script into smaller scripts that perform more atomic parts of the analysis, a common approach for electrophysiology data analysis pipelines. In this scenario, inputs of later scripts are the outputs of earlier scripts in the pipeline (i.e., there is a sequential dependence among the scripts). In our example, *psd_by_trial_type.py* could be broken into two main steps: the first reads an experimental dataset and computes the PSDs for each trial type, and the second creates the plot objects, takes the PSD data from both datasets, plots it using *matplotlib*, and saves the plot as *R2G_PSD_all_subjects.png* (Fig. 14*Aiii*). Although this requires saving the data with the computed PSDs into intermediate files (which adds a file input/output performance cost), the workflow can be orchestrated by management systems such as *Snakemake* that control the parallel or sequential execution of the steps according to the file dependencies (i.e., the PSDs of either monkey N or monkey L can be estimated simultaneously, but the final plotting step must wait for the availability of the PSD data from both subjects). *Snakemake* can distribute the execution of parts of the analysis to specific compute cores and reuses data from previous steps if changes are made to a script in a later step.

As Alpaca tracks the provenance of single-script runs, we implemented the two scenarios described above to demonstrate how to use the tool to track provenance in complex multi-script or parallelization scenarios. Each scenario uses the same functions as the *psd_by_trial_type.py* script described for the use case example, and is instrumented with Alpaca in the same way. Modifications were introduced only to accommodate the requirements for parallelization or breaking the steps into multiple scripts. For MPI, the control flow is modified to process a single subject loop iteration, to plot only on rank 0, and to perform an MPI send/receive operation before the execution of the plotting functions. For *Snakemake*, steps were added to save/load intermediate PSD data as (pickle) files. Each script execution generates an RDF file containing the provenance of that single execution. In the MPI example, 2 RDF files are saved (from rank 0 and 1 executions, respectively). In the *Snakemake* example, 3 RDF files are obtained: two in the step to compute the PSDs (for either monkey N or monkey L) and one in the step for plotting. The data from all RDF files of either MPI (2 files) or *Snakemake* (3 files) examples are trivially combined into a single RDF graph to visualize provenance as a graph. Figure 14*B* compares the graphs obtained for each scenario after aggregation. Due to the unique identifiers generated by Alpaca, when the provenance data of the distributed executions were combined, a fully connected graph describing the whole analysis emerged (e.g., in the *Snakemake* example, the identifiers of the files saved in step 1 are the same when read by step 2). The resulting provenance tracks of the MPI (Fig. 14*Aii*) and the *Snakemake* (Fig. 14*Aiii*) scenarios are highly similar to the single-script scenario (Fig. 14*Ai*), with minor differences due to the changes needed to accommodate the parallelization or multi-script orchestration (e.g., reading additional files).

Therefore, the script-based tracking of provenance using Alpaca can be used in these more complex and distributed scenarios, yielding a merged provenance record that provides the overview of the whole analysis process.

## Discussion

We presented Alpaca, a toolbox to capture fine-grained provenance information when executing *Python* code, with a specific focus on scripts that analyze data. The information is saved as a metadata file that represents a sidecar file to the saved analysis results. Using a realistic use case analysis of calculating power spectra estimates in a massively parallel electrophysiology dataset, we showed how this captured provenance metadata helps in understanding an electrophysiology analysis result that could ultimately be shared among collaborators. With the help of graph visualizations, it is possible to inspect the data flow across functions together with other details that were available at run time, such as object attributes and annotations and function parameters. The toolbox takes advantage of existing standards to represent electrophysiology data in *Python* (e.g., *Neo*) by also capturing relevant object metadata into the provenance records. In the end, it was possible to obtain detailed information that were not available from the result file alone. This provided a better context for the interpretation of an analysis result and adds to the rigor in its reuse.

In the beginning, we introduced four challenges associated with the analysis of electrophysiology datasets that we aimed to consider in designing a toolbox to capture provenance. We then showed, using our concrete use case, that Alpaca addresses these challenges. First, the customized data preprocessing routine using functionality of the *Neo* package was described in the provenance record with all the relevant parameters. Second, any state of the parameters of the functions called in the script and the data flow will be automatically recorded together with the results to allow detailed comparisons as the script is developed and adapted over time. Third, in agile, iterative analysis scenarios, the changes to the source code or execution order of code blocks lead to different result files and to different provenance tracks that can be bound to the result files and code by the file and script identifiers, respectively. Finally, Alpaca provided a structured provenance record describing the history of generation of *R2G_PSD_all_subjects.png* as an additional file that is suitable for sharing together with the results. This serialized provenance makes not only information available in the plot in Figure 3 (e.g., subject names, units) but also that were not apparent at all (e.g., the annotations employed to select the timestamps of the CUE-OFF events that are the start time of the trial data used) accessible in a machine-readable format that can be inspected by scientists receiving the shared analysis results. Overall, the provenance information captured by Alpaca delivers the information required for understanding and interpreting an electrophysiology analysis result, facilitating especially work in collaborative environments.

Trust is a key factor in experimental data analysis, especially in collaborative contexts. Result artifacts (files, figures, etc.) are useful as long as the processes that generated them fit the hypotheses and research questions that guided the analysis in the first place. As provenance information describes the data and its transformations, it is expected that it should help in building trust in the analysis of electrophysiology data. Improving trust in the analysis is one of the focuses of Alpaca, and the provenance information captured as a metadata file helps in that direction. With the example presented in this paper, we demonstrated that the toolbox describes the analysis processes in detail, reducing uncertainty on every step of the data analysis. Data loading, preprocessing, signal processing, obtaining the actual PSD estimates, and preparing the data for plotting and saving the result file were apparent when analyzing the provenance records saved as *R2G_PSD_all_subjects.ttl*. In addition, the key parameters that determine each intermediate result are clearly defined. In the end, Alpaca contributes to building trust in the processing of analyzing data in collaborative environments and sharing results among peers.

Alpaca might improve the reproducibility of the results when analyzing electrophysiology data. Considering reproducibility as the ability to reproduce a given analysis result by different individuals in different settings, the detailed information provided by Alpaca provides a good description of the processes involved in the generation of the analysis result even in the absence of the original script. Although a full re-execution or reconstruction of the source code is neither possible nor the goal of the tool, still it is possible to know the sequence of functions used, their source packages and versions, and the relevant parameters in a level of detail that would help in any reimplementation of the analysis pipeline from scratch. The provided identifiers and hashes would also help in checking whether the data objects are equivalent between runs, without having to serialize the full object data at each step. In the end, although the generation of the exact result file will require the re-execution of the original script, the information summarized by Alpaca already makes any attempts to reproduce the results using a different code more likely to succeed.

Alpaca also contributes to make the electrophysiology data analysis results more compliant to the FAIR principles (Wilkinson et al., 2016). These were developed to provide recommendations and requisites to increase the findability, accessibility, interoperability, and reusability of data. While typically considered in the context of the source data files obtained from an experiment, the principles could be extended to include artifacts such as a result stored in *R2G_PSD_all_subjects.png*. Indeed, increasing the FAIRness of such electrophysiology analysis results would bring several benefits. First, if the results are findable, it is easier to navigate among a collection of results such as hundreds of files in a shared folder. Second, the interoperability would allow for the comparison of similar results produced by different implementations of a single method (such as the case of different *Python* toolboxes providing similar analysis functions, such as the computation of a PSD using the Welch method that is available in *Elephant*, *SciPy*, *MNE* (Gramfort et al., 2013), and many others). Finally, the reusability of the results would eliminate the necessity of repeating required analyses when they were already performed. In the use case presented in this paper, a collaborator might be interested in using the PSD estimates as a starting point for further analyses of the same experimental datasets. If the existing *R2G_PSD_all_subjects.png* already provided an adequate analysis with respect to the preprocessed trial data, signal processing, parameters of the PSD estimates, and aggregation over channels and trials, she could simply reuse it to make any required inferences before starting her analysis. Alpaca provides advances mainly with respect to the reusability FAIR principle, as the analysis results are obtained with detailed provenance, and the results are also described with accurate and relevant attributes such as the annotations present in the *Neo* data objects. However, Alpaca also improves the interoperability and findability of the results. Regarding interoperability, first the provenance information is structured in a machine-readable format, using the PROV provenance model that defines a broadly used vocabulary for provenance representation. Moreover, the metadata (in the form of attributes and annotations of the data objects) and function parameters (that can be seen as a special kind of metadata when considering what is proposed in the FAIR principles) are also structured in a machine-readable format defined formally in the Alpaca ontology. Finally, the findability of the results is improved, as Alpaca binds the identifiers of the individual data objects, files, script, functions, and function executions to the analysis outcome, making it queriable via, e.g., the functions used in generating the outcome or by specific parameter settings. In the end, although a fully FAIR-compliant solution requires the development of additional resources such as controlled vocabularies and ontologies to represent the electrophysiology data analysis processes, Alpaca already provided increased adherence of the electrophysiology analysis result to the FAIR principles.

Besides those improvements associated with the machine-readability of the captured provenance, Alpaca also facilitates the access to the provenance of the analysis results by humans. Facilitating data interpretation is one of the primary focuses of Alpaca. The visualization graphs generated from the RDF files eliminate the necessity of complicated tools such as SPARQL Protocol and RDF Query Language (SPARQL) queries to extract and interact with the captured provenance. This ability to explore the provenance graphs and inspect data object attributes and annotations as well as function parameters allows the scientist to visually understand the details of each individual data transformation which facilitates the interpretation and understanding of the analysis result. This is complemented by the possibility to aggregate similar nodes in the graphs producing summarizations. While these lose the fine-grained details, they provide a high-level overview that is more descriptive of the analysis process than any accompanying textual documentation or the script source code. Ultimately, Alpaca not only records the provenance information for documentation purposes, but helps in understanding and interpreting the analysis result.

Users familiar with graph databases can insert the generated RDF files into triple stores and use the SPARQL query language to introspect the analysis results without relying on the visual graphs. This is an alternative to complement the graph visualizations to obtain more direct answers to specific questions about the provenance of the result (e.g., obtain the list of all distinct functions used to generate a file).

One design feature of Alpaca is that it does not provide a description of the control flow in the script. This is apparent from the main structure of the provenance graph of the example presented in Figure 11*A*, where each iteration of a *for* loop appeared as a separate path starting from the function that generated the objects accessed in the loop. From the implementation perspective, the same graph would be obtained if the source code was structured in a way that the access of individual elements was done without a loop (i.e., instead of looping over a container with N elements, insert N function calls, each using a different element from the container). Therefore, at this point, it is not possible to use the saved provenance to make inferences about the code. In contrast, the data-centric approach taken by Alpaca was developed with the aim of exposing the data and its transformations, and relevant parameters and metadata. Thus, we consider that the resulting provenance lacks complexity while making the data flow clear, regardless of the control flow used to achieve it.

The analysis of electrophysiology data frequently involves more complex workflows than a single script such as the one presented in the example. We demonstrated in Figure 14 that Alpaca can track provenance in multi-script workflows where parallelization is involved. Therefore, the tool is helpful in highly parallelized environments where scripts are frequently used, such as high-performance clusters. The script-based approach could also be useful in cloud-based scenarios where *Python* scripts can be executed, such as Amazon Web Services (AWS) Elastic Cloud instances, or dedicated services for scientific computing, such as *Code Ocean* and *EBRAINS*. Code not implemented in a functional programming style is still poorly supported in this initial version, and this capability is a point to be addressed in future versions of the tool. However, the current functionality is expected to accommodate several typical use cases for analyzing electrophysiology data.

### Comparison with existing tools

There are existing tools that aim to capture and describe provenance during the execution of scripts, and each tool has distinct technical approaches and aims to accomplish distinct objectives (Pimentel et al., 2019 for a review). One approach is to capture provenance during the script run time, as adopted by Alpaca. In this context, we highlight *noWorkflow* (Murta et al., 2015), as it was intended to be used in a similar scenario than Alpaca, i.e., the execution of standalone *Python* scripts that analyze data and produce output files. However, in contrast to Alpaca, *noWorkflow* does not require code instrumentation, but relies on a custom command line tool to run the script. The *noWorkflow* tool performs an *a priori* analysis of the code together with tracing during the script execution to provide a very in-depth description of the sequence of functions called and to generate a detailed call graph as provenance information. All the information is captured and saved in a local database. The focus of *noWorkflow* is storing and describing repeated runs of the code (trials), highlighting the differences and evolution across trials. Although *noWorkflow* provides a very detailed description of the analysis process at the level of every function call (which is not possible for Alpaca as it tracks only the functions identified by the decorator), it falls short for some aspects introduced by Alpaca. First, we decided to save provenance using a data model derived from PROV, which increases interoperability, while *noWorkflow* currently relies on a custom relational database to structure the information on the function executions. Moreover, Alpaca aims to provide an extended description of the data objects across the script execution, which was implemented in the ontology used in the RDF serialization. Together with the description of the sequence of functions executed, this additional information is relevant for the understanding of the analysis result, especially regarding metadata provided as annotations. An example in the presented use case is the identification of the data pertaining to the individual trial types. *noWorkflow* would have shown the loops and sequence of *Neo* functions used to cut the data into the smaller trial segments, but the annotations identifying each *Event* object used for the preprocessing using those functions would not be accessible. In the end, this relevant information is accessible from the provenance records provided by Alpaca. Overall, Alpaca captures provenance with a different perspective on the analysis process, that is more relevant for the particularities of electrophysiology data analysis as introduced at the beginning of this paper.

*AiiDA* (Pizzi et al., 2016) is another tool that can be used to capture provenance in data analysis workflows implemented in *Python*. It was developed as a complete solution for the automation, management, persistence, sharing, and reproducibility of complex workflows. With respect to data provenance, *AiiDA* tracks and records the inputs, outputs, and metadata of computations and produces a complete provenance graph. The technical approach is similar to Alpaca since it also uses decorators to instrument the code. However, *AiiDA* has other design features: (i) it saves provenance in a centralized storage; (ii) as part of the provenance tracking, any data object can be saved to the database with a unique identifier, allowing its retrieval later for reuse together with the lineage. In the end, *AiiDA* is a more holistic tool for reproducibility than Alpaca, as it is possible to re-execute the analysis using the same data objects previously stored. However, we also identify limitations in comparison to Alpaca. First, *AiiDA* requires any existing data objects (such as the ones provided by the *Neo* framework) to be wrapped by custom objects so that the system can identify and serialize their content to the database, which can be achieved through a plugin system. This means that the user must implement this interface for any and every specific data object in a custom framework. This not only requires a considerable amount of effort but this may also introduce a level of maintenance complexity as the data framework evolves and the user needs to ensure that the wrappers retain compatibility in the future. With the approach taken by Alpaca, we tried to keep the original *Python* objects without any fundamental transformation in their structure, and therefore we focused on identifying them using the URNs so that the lineage graph can be constructed, together with the description of their relevant metadata. An additional limitation of *AiiDA* is the overall setup of the system to obtain the provenance information. In the approach taken by Alpaca, the provenance information is saved locally as RDF in an additional file that should accompany the actual results produced by the script, using the interoperable PROV data model. Although sharing the information requires the user to also share the provenance metadata file together, which is less convenient than just querying a database using a command line tool such as the one provided by *AiiDA*, this adds simplicity to use the tool as no special services are required to be set up at the user system. It is important to note that, at this point, the individual RDF files produced by Alpaca could also be stored into a centralized RDF triple store system (either locally or remote) in order to provide similar functionality, if desired. Finally, a third limitation is the use of a non-interoperable standard for description of provenance, as the provenance graphs by *AiiDA* rely on a custom description of the data and control flows, and obtaining the provenance graphs requires the user to query the information using the specific *AiiDA* application programming interface (API) as opposed as using a standard such as SPARQL. In the end, in comparison to *AiiDA*, Alpaca has a reduced entry barrier to implement provenance tracking into existing scripts, which may be relevant for the average electrophysiology lab to start benefiting from provenance capture during the analysis of their experimental data. It is likely that each of the two tools focus on the needs brought by different application scenarios, such as a small lab versus a large research institute. For the small lab, improvements in collaborative work in the analysis of electrophysiology data by capturing more detailed provenance might be quickly achieved by using a tool like Alpaca.

Recently, *CAESAR* (CollAborative Environment for Scientific Analysis with Reproducibility) was proposed as a solution for the end-to-end description of provenance in scientific experiments (Samuel and König-Ries, 2022a). The overarching goal of *CAESAR* is to capture, query, and visualize the complete path of a scientific experiment, from the design to the results, while providing interoperability. This was achieved by the implementation of the REPRODUCE-ME model for provenance (Samuel and König-Ries, 2022b), based on existing ontologies such as PROV-O (Lebo et al., 2013) and P-Plan (Garijo and Gil, 2012). A solution called *ProvBook* is also provided in order to support reproducibility and to describe the provenance of the analysis part of the experiment implemented as *Jupyter* notebooks. Alpaca shares similar concepts with *CAESAR*, as we extended PROV-O to obtain an interoperable description of provenance. However, the provenance information provided by Alpaca is more detailed with respect to the analysis part, which is the main goal of the tool. While *CAESAR*/*ProvBook* provides overall descriptions of changes in the source code of *Jupyter* notebook cells (and the associated results produced by those changes), the details of the functions called inside each cell are not described with the same level of detail as Alpaca. Moreover, although *CAESAR* supports the capture and interoperable serialization of metadata throughout the experiment, Alpaca structures metadata for data objects throughout the code execution during the analysis (e.g., the annotations and attributes of *Neo* objects), which provides a more fine-grained description of the data evolution (e.g., the removal of the two channels from the data from monkey L in the use case example). In the end, *CAESAR* is a useful tool to capture overall aspects of provenance during the execution of an analysis in the context of an electrophysiology experiment. However, the additional level of detail provided by Alpaca is complementary and could be used to provide additional levels to the provenance, while retaining interoperability.

The *fairworkflows* library aims to make workflows implemented within *Jupyter* notebooks more compliant with the FAIR principles (Richardson et al., 2021). The library uses decorators to add semantic information to the *Python* code. After their execution, *fairworkflows* constructs RDF graphs describing the workflows using P-Plan (Garijo and Gil, 2012) and other ontologies defined by the user in the annotations (Celebi et al., 2020). This is linked to the provenance information that is captured during the execution and structured using PROV-O and can be published in the form of nanopublications (Kuhn et al., 2016). The use of decorators to instrument the functions is similar to Alpaca, and the decorators of *fairworkflows* might be used within scripts such as *psd_by_trial_type.py*. However, while Alpaca makes a distinction between inputs, outputs, and parameters (from the arguments that a *Python* function can take and its return values), *fairworkflows* makes a direct mapping of arguments as inputs and function returns as outputs. Therefore, the semantic model for provenance in Alpaca emphasizes the identification of the parameters relevant to control the execution of particular functions. For example, in the computation of the PSD using *welch_psd*, *fairworkflows* would consider the 2 Hz value an input to the function, when Alpaca records it as the special property **hasParameter**. This is particularly relevant when querying the information using SPARQL, for instance. Moreover, Alpaca also captures and describes detailed information about the objects, which we showed to be relevant for the correct interpretation of the results. However, the extra information from the semantic annotations in *fairworkflows* could be combined with Alpaca to provide more descriptive provenance and published using the nanopublication engine.

Computational models are frequently used together with electrophysiology experiments to understand brain function and dynamics. Several state-of-the art simulation engines (e.g., NEural Simulation Tool, Gewaltig and Diesmann, 2007; NEURON, Hines and Carnevale, 1997; Brian, Goodman and Brette, 2008) are available, and many are implemented in *Python* or provide high-level *Python* interfaces where neuronal models with different complexities and biological details can be easily constructed using *Python* scripts (e.g., by using an interface such as PyNN; Davison et al., 2009). In this context, Alpaca might be useful to track the sequence of functions and respective parameters used to instantiate the models in the simulator and run the simulations. This could be used as a complement to tools such as *Sumatra* (Davison et al., 2014), which functions as an electronic lab notebook for simulations, capturing coarse level provenance when executing simulation scripts. Another example is for a tool such as *beNNch* (Albers et al., 2022), which implements a modular workflow for performance benchmarking of neuronal network simulations and could profit from a more fine-grained capture of details in the model and configuration step. Therefore, there is the possibility of also using Alpaca outside of experimental scenarios.

A useful tool for electrophysiology data analysis pipelines is a WMS such as *Snakemake* (Köster and Rahmann, 2012). A particularity of *Snakemake* as a WMS is that it orchestrates the execution of different steps that can take the form of custom *Python* scripts, instead of modular and specific workflow elements such as the ones provided by a WMS such as LONI Pipeline (MacKenzie-Graham et al., 2008). This is attractive when working with electrophysiology data as different aspects of the analysis process (as mentioned in Section 1) can be considered yet providing modular and reusable elements (Gutzen et al., 2024). The *Snakemake* WMS is based on binding input and output files as dependencies to each script executed in sequence. Therefore, one could envision a scenario where a script such as *psd_by_trial_type.py* would have all parameters passed by command line and the execution was controlled by *Snakemake*. In this scenario, *Snakemake* would describe the *NIX* files and the the file *R2G_PSD_all_subjects.png* as inputs and output of *psd_by_trial_type.py*, respectively, together with the description of the command line parameters. However, this would still rely on the correct mapping of all command line parameters to the actual *Python* functions (such as the filter cutoff in *butter* or frequency resolution in *welch_psd*). Any parameters potentially hard coded directly into the function calls would not be captured and would result in a wrong or incomplete description of provenance. In contrast, all function-level parameters are tracked automatically with Alpaca. We successfully demonstrated that Alpaca integrates with *Snakemake*, providing detailed provenance of the operations within the scripts while taking advantage of the WMS orchestration capabilities (Fig. 14). Finally, the provenance description of a *Snakemake* execution in the form of directed acyclic graphs is currently stored in a non-interoperable format. Therefore, Alpaca can be a complementary solution to use with *Snakemake* in more complex analysis scenarios, such as the ones that require multiple scripts. However, the provenance description is enhanced: while the coarse provenance at the file/script level can be provided by *Snakemake*, the additional metadata file produced by Alpaca provides a more fine-grained level of detail regarding each step of the workflow, while adding interoperability.

Alpaca might also complement existing technologies frequently used to analyze electrophysiology data, especially in cloud-based and collaborative environments. *DataJoint* (RRID:SCR_014543; https://datajoint.com) is a database-centered approach to computing and storing analysis results using tailored relational models (Yatsenko et al., 2018). Workflows for the analysis of neurophysiology data can be implemented using *MATLAB* or *Python*-based APIs using reusable and curated components (Yatsenko et al., 2015, 2021). We could expect that Alpaca would track and describe the individual operations performed by the Python objects modeling the underlying database and analyses according to the *DataJoint* framework. However, the challenges of a deeper integration will warrant additional investigation. In addition, *Code Ocean* (RRID:SCR_015532; https://codeocean.com) is a cloud-based service for computational reproducibility, providing the execution environment in containers that integrate code and data into a “compute capsule.” This ensures the reproducibility of the code execution, and the history of the executions is tracked together with the results, all accessible through a Web interface. At this point, the provenance provided by *Code Ocean* will expose details of capsule executions and files produced. In parallel, Alpaca can be used to extract detailed information on the execution inside the capsule’s code. This additional provenance could be linked to the coarse provenance provided by *Code Ocean*. Additional investigation is required to align the provenance information between Alpaca and different execution and workflow environments and database frameworks.

### Limitations

The initial implementation of Alpaca described in this article has some limitations with respect to the scope and visualization of the captured provenance. Here, we describe these and suggest remedies.

First, Alpaca is not capturing and saving information regarding the execution environment such as *Python* interpreter information, packages installed, operating system, and hardware details. However, there are existing tools that can be used for that purpose and that could be used to run a script instrumented with Alpaca (e.g., *Sumatra*; Davison et al., 2014). Moreover, Alpaca could be integrated with such tools to use the information provided by them in the saved provenance records. In the end, we focused on adding granularity instead of reimplementing functionality of existing tools, as this information is more relevant for understanding and sharing the electrophysiology analysis result.

Second, the Alpaca ontology is currently not structured to allow the description of the execution environment. It could be further expanded to include any information regarding the environment, as one could envision a revised Alpaca provenance model and ontology with a PROV Agent subclass that would be related to **ScriptAgent**, and whose properties would describe the relevant aspects of the environment. Moreover, the description could be further improved by integration with other ontologies developed specifically for the detailed description of experimental workflows, such as P-Plan (Garijo and Gil, 2012) and REPRODUCE-ME (Samuel and König-Ries, 2022b). Therefore, although not present in this initial implementation, the approach adopted allows easy expansion and integration of additional features.

Third, some steps are visible from the data flow perspective but they are not fully descriptive and understandable at this point. One example is a user-defined function, such as *plot_lfp_psd* in *psd_by_trial_type.py*. As a plotting function, the user might be interested in knowing additional details on how the inputs (i.e., the *matplotlib* *AxesSubplot* object and the arrays with the data) were handled. The current implementation tracks code in a single scope, and therefore the execution of a function such as *plot_lfp_psd* is treated as a “black box.” It would be interesting to also capture the execution of some functions with an even finer description of the operations inside those functions. This could be achieved by expanding the functionality to automatically include functions in levels lower than the primary capture scope. However, even in the current implementation of Alpaca, although such fine descriptions from inside of *plot_lfp_psd* are not available, the provenance stored in the generated metadata file already points to where the function was implemented. In this way, the user can focus on inspecting the implementation of the function *plot_lfp_psd* and does not have to check the full source code.

Fourth, only a generic visualization graph is currently provided in Alpaca. The initial version of Alpaca is intended to provide the basic model and functionality to capture and describe provenance when analyzing electrophysiology data while providing essential visualization. Although we took the approach to leverage the advantage of open source graph visualization tools such as *Gephi*, the visualization of the captured provenance is not optimized (e.g., showing only parameters of the selected function or object). Such optimized visualization can be incorporated as additional feature in Alpaca without any changes to the captured information or serialization as RDF, by using existing graph visualization frameworks such as *Pyvis* to build a customized visualization environment based on the information in the RDF graphs and the Alpaca provenance model. Finally, there are existing tools that specifically deal with the visualization of provenance graphs. One example is *AVOCADO* (Stitz et al., 2016), implemented to be an interactive provenance graph visualization tool that exploits the topological structure of the graph to provide a visual aggregation. Although Alpaca provides basic aggregation using functionality adapted from *NetworkX*, we could also leverage a tool like *AVOCADO* to provide visualization functionality more tailored to the features of a provenance graph, such as hierarchical structure (e.g., all the steps in a single-trial-processing loop grouped in a single node) and temporal evolution (isolating the visualization of the analyses performed in the first or the second dataset). However, the technical challenges of such integration are unknown at this point.

Fifth, although the design of Alpaca allows capturing and describing any *Python* object used by a function, the serialization of extended details according to the Alpaca PROV model (i.e., attributes and annotations) is currently limited to *NumPy*-based objects such as *NumPy* arrays, *quantities* arrays, and *Neo* objects. With this initial version of Alpaca, we aimed to establish the foundational capabilities to describe data object metadata in the captured provenance, as this is an essential feature to understand and interpret the analysis result, without focusing on extensive coverage of the data models currently available in *Python*. It is important to mention that the functionality to describe the data objects in detail is already implemented as a plugin system, where a *Python* package can insert a specific function to fetch information from objects used by that package. Therefore, support for capturing detailed information besides those selected cases (e.g., *NWB* or *Pandas DataFrames*) can be achieved by implementing the relevant function for the package and adding a new interface for the user to define attributes of a particular object to be captured.

Finally, Alpaca does not allow rerunning the code to reproduce the analysis result fully. This was not the focus of the tool, and such functionality could be achieved by integrating with existing tools that allow code re-execution. One candidate is *Sumatra*, as it not only captures the information on the environment but also allows re-executing the script with the same parameters as the original run. Moreover, we demonstrated that Alpaca can easily integrate with a script-based WMS such as *Snakemake* that supports re-executing the code. Rerunning the analysis can also be accomplished within systems that control script execution, such as *Code Ocean*. In the end, any existing tool that properly manages environment management and script invocations might be used to rerun the code, while Alpaca adds an additional level of detail to the captured provenance aimed at increasing interpretability.

### Future directions

Several improvements are planned for Alpaca in the future. First, we plan to expand the toolbox to also capture provenance for analyses implemented using *Jupyter* notebooks. Not only is *Jupyter* extensively used for exploratory data analysis, but also the repeated execution of code cells and subsequent substitution of data objects in memory requires detailed provenance tracking for reliable description of any analysis result produced by a notebook.

Also, the provenance records lack semantic information that are relevant for understanding electrophysiology data and metadata. Therefore, a further improvement is to allow the inclusion of classes and vocabularies defined in domain-specific ontologies in the provenance records, which will bring further improvements to the FAIRness of electrophysiology analysis results. Using semantic information will improve the interpretation of the captured provenance by scientists unfamiliar with the script code and toolboxes used in the analysis. For instance, the graph visualizations could be improved with this information to display a human-readable, programming language independent label defined in the ontology class instead of the function names defined in the *Python* code. This would help understand steps using functions defined in analysis toolboxes (e.g., *Elephant* and *Neo*) and user-defined functions, whose understanding requires referring to the original code. This would also allow an easier assessment of differences and similarities when comparing provenance from different analyses and further simplify understanding the provenance outside the context of the original code.

The functionality will also be improved to capture information about the execution environment, together with information from version control systems such as *git*, to provide more detailed information about the source code that originated the analysis result. Planned improvements include automatically capturing information on the *Python* interpreter, operating system and hardware, and details of the *Python* packages where the functions are implemented (cf., e.g., *Sumatra*).

Furthermore, we propose to integrate a specific tool to aid in comparing different provenance files to facilitate identifying differences between analyses. The goal is to leverage information provided by the provenance model implemented by Alpaca, especially the metadata captured as attributes and annotations, in order to help scientists draw informed conclusions based on differences among a set of results.

We aim to further improve the interaction and analysis of the captured provenance by developing a custom visualization and search interface based on the serialized RDF graphs. This tailored visualization interface is planned to be aware of the provenance model implemented in Alpaca, and use more user-friendly resources such as floating labels to show annotations and attributes of the data or function parameters, or interactive visualization controls such as graph expansion/aggregation on demand.

Finally, we aim to investigate how the captured provenance can be integrated with existing tools in the neurophysiology data ecosystem. A potential integration is how to incorporate the generated provenance metadata into standards to share neurophysiology data, such as *NIX* and *NWB* file formats. Files written using these standards could easily embed the RDF files or their information as metadata. In addition, Alpaca could be integrated with *Python* packages used in the manipulation, preprocessing, and analysis of electrophysiology data (e.g., *Neo*, *SpikeInterface*, *Elephant*) to provide embedded provenance capture functionality, eliminating the requirement for the user to instrument functions from packages that are frequently used.

### Conclusions

We implemented Alpaca, a toolbox for lightweight provenance capture during the execution of *Python* scripts used for the analysis of electrophysiology data. Alpaca captures more detailed information about the analysis processes, including not only the lineage of the data but also embedded metadata relevant for the description of data objects during the processing pipeline. In the end, this makes the electrophysiology analysis result artifacts more compliant to the FAIR principles. This may improve research reproducibility and the trust in the results, especially in collaborative environments. Therefore, Alpaca may be a valuable tool to facilitate sharing electrophysiology data analysis results.

## Synthesis

Reviewing Editor: Arvind Kumar, KTH Royal Institute of Technology Decisions are customarily a result of the Reviewing Editor and the peer reviewers coming together and discussing their recommendations until a consensus is reached. When revisions are invited, a fact-based synthesis statement explaining their decision and outlining what is needed to prepare a revision will be listed below. The following reviewer(s) agreed to reveal their identity: Oliver Rübel, Luiz Tauffer.

Synthesis

The two reviewers who read your manuscript agree that the manuscript addresses an important and challenging problem. The PYTHON package Alpaca,is designed to standardize and facilitate the tracking of provenance information using the data analysis scripts. This is more directed towards experimentalists.

However the reviewers have also raised some concerns that need to be addressed.

One of the main limitations of the packages is that it does not support rerunning analyses, that means that the focus appears to be on facilitating data interpretation and trust. The provenance information seems to be interpretable only in the context of original code and therefore it may not be useful to those who did not generate/analyzed the data. Reviewers have also raised a few more pertinent points - as you will see at the end of this message.

I would like to invite you to revise your manuscript according to the points raised by the authors.

Reviewer #1

Overall, the manuscript is written well and easy to follow. The authors present a balanced assessment of the Alpaca tool and its application to electrophysiology data.

The manuscript targets an interesting and highly challenging problem of data provenance for data analysis. The approach described in the paper, and implemented in the Alpaca software, emphasizes a lightweight approach in terms of instrumentation and depth of provenance data that appears geared towards experimental neuroscientists rather than data science professionals. Making solutions accessible to lab scientists is an important need. While the approach taken is sound, it has several key limitations that appear to currently limit its utility in practice.

Capturing information about the provenance of analyses can have many different goals, among others e.g.: i) rerunning an analysis to replicate results, applying to new data, or changing parameters, ii) supporting interpretation of analysis results by supporting introspection of the analysis process, iii) improving transparency and trust. While the tool provides an interesting and relevant step towards making data provenance more accessible, its utility in practice is not fully clear. The tool does not support rerunning analyses, so the focus appears to be on facilitating data interpretation and trust. However, the provenance information captured appears to be interpretable only in the context of original code used to execute an analysis. As such, the provenance appears useful to the scientists that generated the analysis but not easily usable for others. The mechanisms available for introspection of the results appear limited to static graph-based visualization, which are not easy to use for the target audience.

From a technology perspective the tools appear limited to analysis scripts designed in a functional programming style and executed in a monolithic fashion, limiting the application of the tool in practice. Application in parallel and cloud-based analysis environments is not being discussed. Application to modular workflows (consisting of many scripts) is not clearly discussed. The authors mention interactive changes of parameters, however, the tool does not appear amenable to that use case, but rather target the re-execution of analysis with varying parameters. In terms of inputs, the tool appears closely tied to the NEO/NIX tools, and it should be clarified how other data sources are being handled.

In terms of comparison to other tools, the author presents overall a fair and detailed assessment. The authors appear to place the tool somewhere in-between deep and shallow provenance technologies to fill a gap in accessibility, which is viable and important. In the area of electrophysiology other highly relevant technologies are DataJoint (which employees a pipeline- and database-driven approach to technology) used, e.g., by the International Brain Lab, and container-based technologies (e.g., Codeocean), used e.g., by the Allen Institute for Neural Dynamics. Since the manuscript targets electrophysiology it would be useful to target the comparison not just broadly to tools, but to the approaches currently employed in the application domain. Overall, the tool appears helpful to increase transparency in environments where best data analysis and coding practices are not implemented and provides a reasonable step towards improving practices.

From a computer science perspective, the tool presents only incremental improvements to alternate systems. As such, the main contribution of the manuscript appears to be towards enhancing provenance practices in the specific application domain.

Reviewer #2

The manuscript describes Alpaca, a new Python package aimed at standardizing and facilitating the tracking of provenance information, with focus on neurophysiology data analysis scripts. While similar tools exist for similar purposes, the authors highlight the relevant differences between these tools and Alpaca, that makes the latter better suited for the task. Provenance recording and standardization is currently a relevant issue, and the manuscript provides advances in that area.

There are a couple of points which I feel could be addressed to benefit the article. I'm putting them together with other notes that might be valuable for the software but were not mentioned in the manuscript:

1. The tool seems to be general enough to be used for any analysis script, not only electrophysiology analysis. But, if the authors want to focus on electrophysiology, e.g. for automatically extracting specific objects properties, why not have this as an extension or plugin of established tools such as SpikeInterface (which uses Neo as a IO backend but offers a much wider variety of processing options)?

2. Why is a RDF representation needed here? Wouldn't any directed acyclic graph representation be enough to cover the level of provenance tracking proposed by this tool? Could the authors propose any advantage of using a RDF representation for provenance tracking, e.g. a network analysis for a series of experiments? It is not immediately clear how the semantic layer brought by RDF can be exploited in this context.

3. One of the most relevant aspects of provenance should be the execution environment. If constrained to Python functions alone, it would be very easy to automatically include at least Python and packages versions. In the Limitations session the authors explain their focus was on extending the provenance within scripts, which is good, and that it would be easy to integrate the environment or system level information provided externally by other tools. I suggest the authors consider automatizing this step in future features of the software.

4. The distinction between Alpaca and workflow management systems such as Snakemake was well highlighted. In later iterations of the software it would be interesting to see examples where both tools can be used complementarity, since real life analysis pipeline are more often multi-script.

5. It is not clear if the Alpaca tool has a diff method, which would help identifying differences between two provenance files. This would be one of the main advantages of incorporating Alpaca in analysis pipelines.

6. the authors comment that storing provenance information as an extra file might be mildly inconvenient for sharing. This shouldn't be taken as a disadvantage of their approach, since modern and powerful ways of sharing neurophysiology data, such as the Neurodata Without Borders, can easily incorporate the provenance metadata within the same file. I suggest the authors consider developing basic integration between Alpaca and such data standards, it would be valuable for the neurophysiology data sharing ecosystem.

## Author Response

Comments from the Editor Manuscript Instructions - Supplemental material is not allowed. This change can be made later if your paper receives a revise decision. We removed the supplement, which consisted of a piece of explanatory code, and stored this information as PDF in the online repository for the code reproducing the manuscript figures under the file name code_changes.pdf. The full link is provided in the text (page 7, lines 314-315). It is redacted in the version highlighting changes for the double-blind review, and available in the clean version with the full title page. Synthesis of Reviews The two reviewers who read your manuscript agree that the manuscript addresses an important and challenging problem. The PYTHON package Alpaca is designed to standardize and facilitate the tracking of provenance information using the data analysis scripts. This is more directed towards experimentalists. However the reviewers have also raised some concerns that need to be addressed. One of the main limitations of the packages is that it does not support rerunning analyses, that means that the focus appears to be on facilitating data interpretation and trust. The provenance information seems to be interpretable only in the context of original code and therefore it may not be useful to those who did not generate/analyzed the data. Reviewers have also raised a few more pertinent points - as you will see at the end of this message. I would like to invite you to revise your manuscript according to the points raised by the authors. We would like to thank the reviewers and the editor for their insightful and helpful comments. We responded to all the raised concerns point-by-point below. We believe that we have addressed the pertinent points of both reviewers. In particular, we clarified how the provenance information recorded by the Alpaca tool and its underlying data model can be meaningfully interpreted by those who did not generate or analyze the data. All changes to the text have been highlighted in the revised version of the manuscript. Reviewer #1 Overall, the manuscript is written well and easy to follow. The authors present a balanced assessment of the Alpaca tool and its application to electrophysiology data. The manuscript targets an interesting and highly challenging problem of data provenance for data analysis. The approach described in the paper, and implemented in the Alpaca software, emphasizes a lightweight approach in terms of instrumentation and depth of provenance data that appears geared towards experimental neuroscientists rather than data science professionals. Making solutions accessible to lab scientists is an important need. While the approach taken is sound, it has several key limitations that appear to currently limit its utility in practice. We thank the reviewer for the detailed and inspiring review of our manuscript, and in particular the comments on the current limitations. We will address all the points with the replies below. All changes to the text have been highlighted in the revised version of the manuscript. Capturing information about the provenance of analyses can have many different goals, among others e.g.: i) rerunning an analysis to replicate results, applying to new data, or changing parameters, ii) supporting interpretation of analysis results by supporting introspection of the analysis process, iii) improving transparency and trust. While the tool provides an interesting and relevant step towards making data provenance more accessible, its utility in practice is not fully clear. The tool does not support rerunning analyses, so the focus appears to be on facilitating data interpretation and trust. The reviewer is fully correct in the assessment that the main goal of Alpaca is to facilitate data interpretation and trust. We would perhaps add a third aspect, which is to aid the findability of a derived data asset by means of a search of accompanying provenance information, e.g., for a provenance trail containing a certain data file and a certain analysis function. Several tools are available to support rerunning the analyses (e.g., Sumatra for recording provenance of a computational run, or Snakemake as a workflow management tool), so our approach was to emphasize the description of the data flow, which is information that cannot be fully inferred from the underlying code alone. In this way, we reasoned that the results from the analysis process can be understood with respect to relevant aspects that affect its interpretation (e.g., confirming which recording channels were removed in the preprocessing, or inspecting the exact time intervals and behavioural parameters of each experimental trial included in the analysis). We also would like to point out that although Alpaca does not allow rerunning the analysis and, contrary to a tool such as noWorkflow that provides a detailed description of individual microscopic operations performed by the code without tracking data flow between these, Alpaca operated on an intermediate level of granularity (or, detail) of the data flow that aims to makes the essential data operations, data properties and parameters understandable by others. Therefore, our primary goal with the provenance information was to balance the level of detail in the introspection to provide a birds-eye view of the analysis process that facilitates the interpretation of the results. We modified the Discussion, where we comment on trust in the analysis (page 17, lines 760-761), supporting interpretation (page 17, line 754, and page 18, lines 815-816 and 825-826), and limitations (page 23, lines 1084-1093) to make this clear. However, the provenance information captured appears to be interpretable only in the context of original code used to execute an analysis. As such, the provenance appears useful to the scientists that generated the analysis but not easily usable for others. We apologize if this is the impression that was conveyed by our manuscript. Indeed, one of the main goals of Alpaca is to pave the road to obtain understandable descriptions of an analysis process without the need for code review. It is true that a prerequisite for this to work is that the code follows good coding practices (e.g., function names, parameters and variable names must be telling, doc strings are provided,...). For this, it is helpful that a user inspecting a provenance trail is familiar with the used libraries to better understand the steps identified by function names (e.g., preprocessing functions of the Neo library), and that user-defined functions are sensibly named. Using this knowledge, we argue that it is still possible to infer major steps and decisions in the analysis by inspecting the graph without knowing the script. We illustrate this point by giving the following new example, for which we purposefully do not provide the code: In this figure, two plots with the same name were produced by different versions of a script to compute a power spectral density (PSD) for every trial in one of the Reach2Grasp datasets, but varying only the function that computes the PSD. A scientist may notice that the two plots are different and be curious as to where the difference is. The provenance graph for each plot is shown below each plot and the nodes are colored according to the label. It is apparent that the structure of the graphs is similar, but comparing node by node, the difference between the welch_psd and the multitaper_psd nodes is evident. The upper plot is computed using Welch, and the lower using the multitaper PSD method (as the function names hint about the method used in each plot). Therefore, the scientist would already know the major source of difference between the plots without any prior knowledge of the code or of the details of each function used. In more complex examples, in a similar manner of inspecting the graphs, a user may also detect different compositions of functions, differences in parameters or differences in the loaded data. The reviewer is right in case one does not use standard library functions (e.g., user-defined functions such as plot_psds), where the interpretation of such a step without knowing the original script may be more difficult. Therefore functions must have telling names to easily infer the functionality from the captured provenance trace (i.e., a graph node named my_function in the plotting step would be incomprehensible, as opposed to a node labeled load_data). But even in this case it is still possible to identify the source of the function and, as we point out in the discussion on limitations (from page 22, line 1041 to page 23, line 1053), this already provides useful information to the user reviewing the provenance information as she can directly refer to the implementation of that specific function even if not familiar with the whole script. We point out that, to further overcome those limitations, new functionality implemented in the next release of Alpaca allows the definition of semantic information for the captured functions and data objects (e.g., by defining an ontology class that represents the process implemented by a function). In this way, a node may be labeled not only by a function name, but by a semantic expression referring to a specific type of operation (e.g., "Compute Power Spectral Density Multi-Taper" instead of "multitaper_psd"). This could be used in the visualization/disambiguation, facilitating the understanding by scientists not familiar with the code, and helping in those cases. We made this point more clear in the Discussion (page 24, lines 1104-1112). Finally, the provenance captured by Alpaca describes the data flow rather than the process flow and captures information on the data's runtime state. This is the case, for example, for the number of elements returned by the cut_segment_by_epoch function. Such information is now apparent to a scientist reviewing the provenance (shown as the multiplier numbers in the graphs in Figure 12, which are available after the graph aggregation). The number of Segment objects would not be known even if the scientist got familiar with the code. Therefore, by just reviewing the detailed provenance it is possible to infer that a single piece of data of longer duration was split into N smaller chunks, which is a typical step in preprocessing electrophysiology data for analysis, regardless of being familiar with the implementation details. The mechanisms available for introspection of the results appear limited to static graph-based visualization, which are not easy to use for the target audience. The reviewer is right. However, the lack of dedicated tools for easy visualization of provenance information is a general problem, especially when graphs become increasingly complex. This is the reason for initially proposing the use of a general graph visualization tool such as Gephi. Although the Gephi interface requires a learning curve from the user, it is possible to visualize a large graph easily and there are options for her to inspect and filter the data. We fully agree that the next useful step is to provide a tailored visualization interface that is aware of the PROV model in general, and the PROV implementation of Alpaca in particular, using more user-friendly resources such as floating labels to show annotations and attributes of the data or function parameters, or interactive visualization controls such as graph expansion/aggregation on demand. However, it is a major endeavor to develop such an application and this was not the scope of the initial version of Alpaca, where we wanted to implement the foundation to capture and describe provenance while providing essential visualization capability. We emphasized these remarks on the visualization in the Discussion (see paragraphs on page 23, lines 1054-1063, and page 24, lines 1124-1129). We also would like to point out that, although the initial release does not provide this specific functionality, the RDF data can still be loaded in a triple store graph database to create a knowledge graph that allows querying the provenance to answer specific questions (like the example with multitaper vs. Welch method that we presented in the answer above). Therefore, this is an alternative to a static graph-based inspection, and enhanced systems to facilitate querying the information might be an additional development in the future. We also added this remark to the Discussion, to emphasize this other way of introspecting the results (page 18, lines 827-831). From a technology perspective the tools appear limited to analysis scripts designed in a functional programming style and executed in a monolithic fashion, limiting the application of the tool in practice. Application in parallel and cloud-based analysis environments is not being discussed. Application to modular workflows (consisting of many scripts) is not clearly discussed. It is true that we have that type of script in mind. The support for different styles of programming besides functional programming requires additional development, and this is an improvement planned for future versions. Nevertheless, complex workflows consisting of sequences of multiple scripts (such as workflows orchestrated by workflow managers like Snakemake) typically already provide a broad description of the provenance during the orchestrated execution, and we actually expected Alpaca to provide an additional level of detail within each script execution in the workflow. As we agree that this is an important point (also raised in a similar manner by the second reviewer), we included an additional section and figure to the results (from page 15, line 671 to page 16, line 723, and Figure 14) to illustrate how the tool is compatible with these scenarios. Briefly, the single monolithic script is broken into a parallelized version with in-memory data transfer using MPI in a first scenario, and into a multi-script workflow using Snakemake in a second scenario. Despite slight differences related to the requirements of each implementation (e.g., having to save/load data from intermediate files when using Snakemake), the provenance graphs overlap in the main steps. This is achieved as each single RDF graph produced by a single script run can be trivially merged and connected using the identifiers. Therefore, there is room for flexibility in how the analysis is implemented, to accommodate common scenarios. We would like to point out that monolithic scripts are common when using high-performance computing clusters and queuing systems such as SLURM. As several analyses may use methods that require extensive computational power, this is the main reason for the script-based approach. For analyses in a cloud environment, the execution of a script instrumented with Alpaca would be indistinguishable, and we expect that Alpaca would work with little effort (i.e., if the script from the presented example would be run in a service such as Amazon Elastic Compute Cloud or a dedicated system such as Code Ocean/EBRAINS, the provenance would still be captured). For more specific cloud-based services, this would require a special integration. We added these points to the Discussion as well (page 18, lines 844-853). The authors mention interactive changes of parameters, however, the tool does not appear amenable to that use case, but rather target the re-execution of analysis with varying parameters. The reviewer is correct, and we apologize for the misunderstanding. Indeed, what we meant is re-executing analyses with varying parameters in an iterative scenario. Therefore, we changed instances in the text to refer to the word "iterative" instead of "interactive". This includes, e.g., the abstract and the first paragraph describing the challenges for provenance capture (page 3, line 115), and the caption of Figure 1. We would like to point out that we are currently extending Alpaca and the provenance model to capture and describe provenance within Jupyter notebooks, which will also help for the description of provenance in those iterative scenarios and may allow capturing provenance in a full interactive scenario where the user changes the parameters using interactive controls. In terms of inputs, the tool appears closely tied to the NEO/NIX tools, and it should be clarified how other data sources are being handled. While Alpaca works with any Python object, the reviewer is absolutely correct that extended details (e.g., object annotations) are currently defined for Numpy-derived objects such as Neo/NIX. This was implemented with a plugin approach in mind to allow easy expansion and support of additional frameworks. Therefore, adding support to another framework as a data model requires implementing a plugin function to properly serialize the object metadata to RDF. We plan to include more plugin functions in new releases. We point out that, for other common data objects such as Pandas DataFrames, Alpaca can already capture basic attributes such as shape and data type. We made an addition to the description of the tool (page 9, lines 379-380 and Table 1) and the Limitations part in the Discussion (page 23, lines 1072-1083) to describe the availability of detailed information currently only for selected cases. In terms of comparison to other tools, the author presents overall a fair and detailed assessment. The authors appear to place the tool somewhere in-between deep and shallow provenance technologies to fill a gap in accessibility, which is viable and important. Yes, we agree. Between shallow and deep was the initial goal when developing Alpaca. We hope that the responses to the queries above further illustrate this point, and thank the reviewer for the encouraging comment. In the area of electrophysiology other highly relevant technologies are DataJoint (which employees a pipeline- and database-driven approach to technology) used, e.g., by the International Brain Lab, and container-based technologies (e.g., Codeocean), used e.g., by the Allen Institute for Neural Dynamics. Since the manuscript targets electrophysiology it would be useful to target the comparison not just broadly to tools, but to the approaches currently employed in the application domain. We added an additional paragraph to the section comparing tools in the Discussion (see from page 21, line 1003 to page 22, line 1019). Overall, the tool appears helpful to increase transparency in environments where best data analysis and coding practices are not implemented and provides a reasonable step towards improving practices. We thank the reviewer for the encouraging comment. From a computer science perspective, the tool presents only incremental improvements to alternate systems. As such, the main contribution of the manuscript appears to be towards enhancing provenance practices in the specific application domain. While we build on the well-established principles of provenance capture developed in computer science, we would argue that we fill a niche by proposing a conceptual idea of capturing provenance at a mid-grain level, which enhances provenance practices in electrophysiology data analysis. From a research perspective, we believe Alpaca can help to study how the findability and comprehensibility of computationally derived data can be enhanced by enabling descriptions on this level. Reviewer #2 The manuscript describes Alpaca, a new Python package aimed at standardizing and facilitating the tracking of provenance information, with focus on neurophysiology data analysis scripts. While similar tools exist for similar purposes, the authors highlight the relevant differences between these tools and Alpaca, that makes the latter better suited for the task. Provenance recording and standardization is currently a relevant issue, and the manuscript provides advances in that area. We thank the reviewer for the valuable feedback and helpful comments. We will address all points with the replies below. All changes to the text have been highlighted in the revised version of the manuscript. There are a couple of points which I feel could be addressed to benefit the article. I'm putting them together with other notes that might be valuable for the software but were not mentioned in the manuscript: 1. The tool seems to be general enough to be used for any analysis script, not only electrophysiology analysis. But, if the authors want to focus on electrophysiology, e.g. for automatically extracting specific objects properties, why not have this as an extension or plugin of established tools such as SpikeInterface (which uses Neo as a IO backend but offers a much wider variety of processing options)? The reviewer is correct that Alpaca is general enough to capture the provenance of analysis scripts from other domains. Alpaca was designed as a stand-alone tool to enable its integration with various user scripts and toolboxes (i.e., Python packages), thereby simplifying maintenance and reducing dependencies (for both sides). Our design perspective is that for any toolbox, in particular electrophysiology-related tools (e.g., SpikeInterface, Elephant, Neo,...), even without touching the package of the original tool, one can provide an alternative installable PyPI package that is provenance enabled (i.e., where Alpaca is a requirement and where the decorator is applied to the relevant functions exposed by the toolbox API). In this way, the core functionality of provenance capture by Alpaca on the one hand, and the specialization to particular functions or data types in a particular context on the other, are kept separate. Alpaca also supports extraction of extended metadata from special data types (such as the Neo object model in our example) in a plug-in type manner, further simplifying the task of making Alpaca aware of a particular code. We have preliminary work in this direction for the Elephant toolbox. We added this remark to the Discussion on limitations (page 23, lines 1072-1083) and future improvements (page 24, lines 1130-1137). 2. Why is a RDF representation needed here? Wouldn't any directed acyclic graph representation be enough to cover the level of provenance tracking proposed by this tool? Could the authors propose any advantage of using a RDF representation for provenance tracking, e.g. a network analysis for a series of experiments? It is not immediately clear how the semantic layer brought by RDF can be exploited in this context. The reviewer is right that any directed acyclic graph representation would suffice to represent the data flow, similar to how it is presented in the figures of the manuscript. However, the semantic layer of RDF provides advantages when trying to analyze the provenance or to compare provenance information. Specifically, RDF allows using knowledge representation and reasoning technologies such as OWL to make inferences on the graphs. With that in mind, we recently included a new feature in Alpaca to annotate the base nodes of the RDF representation used by Alpaca (DataEntity and FunctionExecution) with additional classes defined in one or more ontologies. With this feature, the generic provenance semantics (i.e., "a function execution F uses data X and generates data Y") can be enriched with information specific to the (electrophysiology) analysis domain (e.g., "the function execution F is a computation of a power spectral density (PSD) using the Welch method, uses the time series data X and generates the PSD data Y"). With available reasoners, it is easy to derive information using the axioms defined in the ontology (e.g., whether function F is a power spectral density computation, regardless of the algorithm). The ultimate goal is to support network analyses such that the provenance of different analyses can be more easily understood with respect to similarities and differences of the steps involved. We added this remark to the part in the Discussion where we comment on the use of semantic information (page 24, lines 1104-1112). 3. One of the most relevant aspects of provenance should be the execution environment. If constrained to Python functions alone, it would be very easy to automatically include at least Python and packages versions. In the Limitations session the authors explain their focus was on extending the provenance within scripts, which is good, and that it would be easy to integrate the environment or system level information provided externally by other tools. I suggest the authors consider automatizing this step in future features of the software. We thank the reviewer for the remark. Indeed, the automatic capture of the environment is a goal, and we plan to leverage existing tools wherever possible. We expanded the paragraph to the "Future directions" section, where we presented this idea, to make this goal explicit. Please, see page 24, lines 1115-1118. 4. The distinction between Alpaca and workflow management systems such as Snakemake was well highlighted. In later iterations of the software it would be interesting to see examples where both tools can be used complementarity, since real life analysis pipeline are more often multi-script. As this was also a point from the other reviewer, we expanded the example presented in the paper to practically demonstrate how a multi-script WMS such as Snakemake can be used together with Alpaca. We added a new section to the Results (from page 15, line 671 to page 16, line 723) and Figure 14. The central idea is that Alpaca is able to obtain the full provenance starting from the input file to the output file of a given rule (i.e., workflow step) in Snakemake. As the input/output files can be uniquely identified, the full provenance track is established by combining the multiple RDF graphs generated by each execution of the script associated with a Snakemake rule. This is possible because when a file output by a rule is the input of the next rule, both nodes will share the same identifier based on the SHA256 hash. 5. It is not clear if the Alpaca tool has a diff method, which would help identifying differences between two provenance files. This would be one of the main advantages of incorporating Alpaca in analysis pipelines. Currently, Alpaca is focused on the capture of provenance, not on the analysis of provenance (such as a diff). In fact, such graph analysis is challenging, both computationally as well as from a user experience. We have started preliminary work on two distinct approaches to identify differences in provenance graphs, specifically tailored to enhance the comprehension of electrophysiology data analysis. The first approach employs a similarity measure to analyze a simplified version of the graphs, considering only nodes (excluding edges) to avoid an exponential increase in computational demands. This allows us to discern differences while taking into account relevant metadata (e.g., channel names) or function parameters, but ignores the order of execution and the data flow. Such a comparison can be used, e.g., to identify putative groups of similar provenance tracks in a larger collection. The implementation can be implemented using graph similarity measures, such as the graph edit distance, which are implemented in the networkx Python library. The second approach utilizes a text-diff-based methodology to pinpoint changes in the order of function executions or the introduction/removal of functions in a provenance track. To implement such an approach, nodes are converted to text strings, using special rules to govern how in- and out-degrees > 1 are to be handled. However, such a straightforward approach is challenging when the two provenance tracks in the comparison structurally diverge. In this case, advanced methods and visualizations are required to identify similar subgraphs to particularly handle the complexity of provenance graphs. Our continued research and development involve developing an efficient provenance graph analysis tool, which aims to benefit the inspection of provenance graphs in general, independent of Alpaca. We added a comment in the Discussion, where we present future directions, to make this goal clear to the reader (see page 24, lines 1119-1123). 6. The authors comment that storing provenance information as an extra file might be mildly inconvenient for sharing. This shouldn't be taken as a disadvantage of their approach, since modern and powerful ways of sharing neurophysiology data, such as the Neurodata Without Borders, can easily incorporate the provenance metadata within the same file. I suggest the authors consider developing basic integration between Alpaca and such data standards, it would be valuable for the neurophysiology data sharing ecosystem. We thank the reviewer for the remark. We incorporated the suggestion and made changes in the Discussion to present it in a less negative phrasing (see page 20, lines 905-909). We also added a paragraph in the section describing future improvements, mentioning the integration with existing standards (see page 24, lines 1130-1137).
