# Supplementary figures and images for: Facilitating the Sharing of Electrophysiology Data Analysis Results Through In-Depth Provenance Capture
Source: eNeuro. 2024 Jun 7;11(6):ENEURO.0476-23.2024. doi: 10.1523/ENEURO.0476-23.2024 (PMC11181106; doi:10.1523/ENEURO.0476-23.2024)

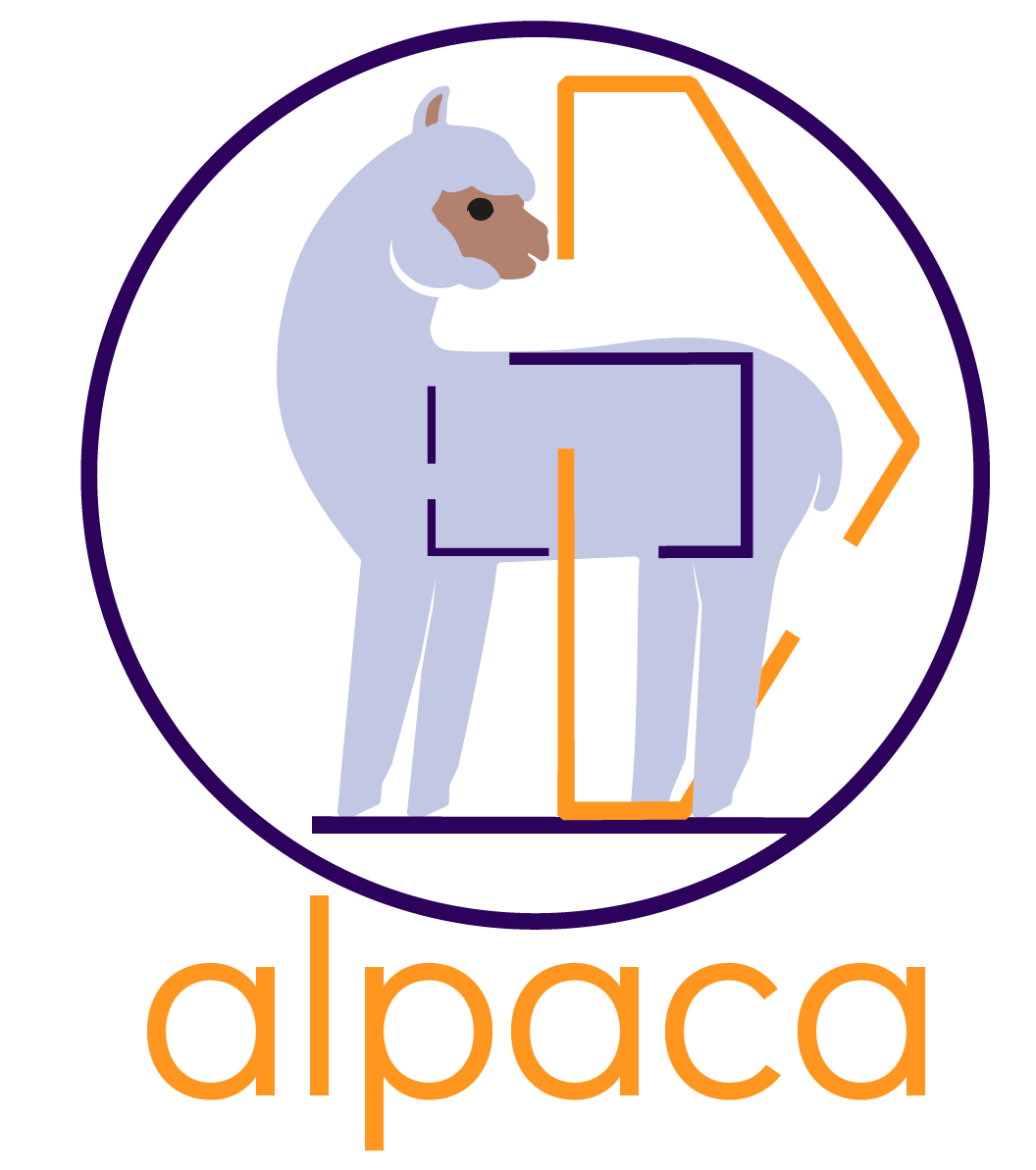

Supplement: Extended Data 1 — Download Extended Data 1, ZIP file. [file eneuro-11-ENEURO.0476-23.2024-s001.zip › doc/_static/images/alpaca_logo.png]

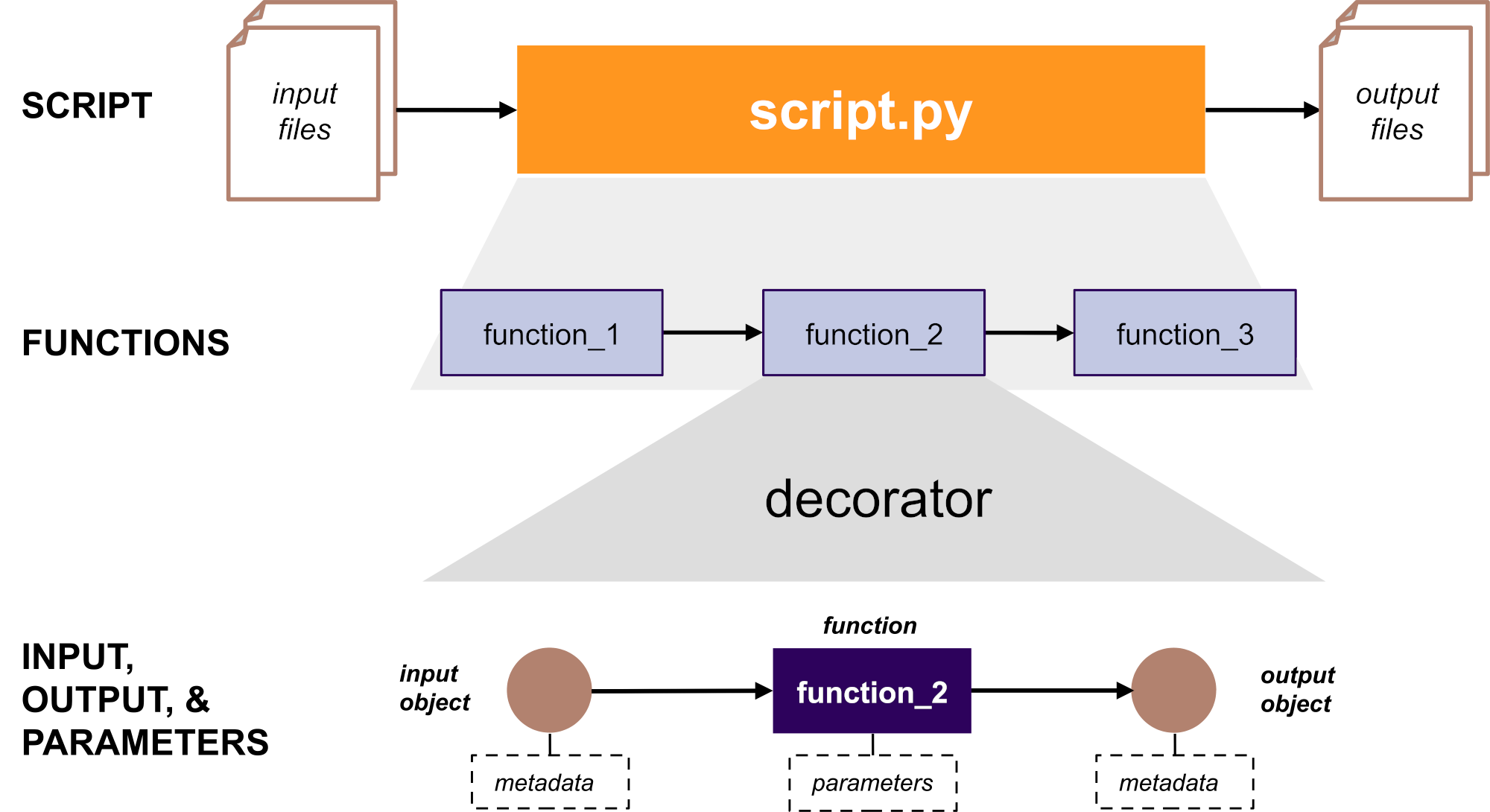

Supplement: Extended Data 1 — Download Extended Data 1, ZIP file. [file eneuro-11-ENEURO.0476-23.2024-s001.zip › doc/_static/images/alpaca/alpaca_decorator.png]

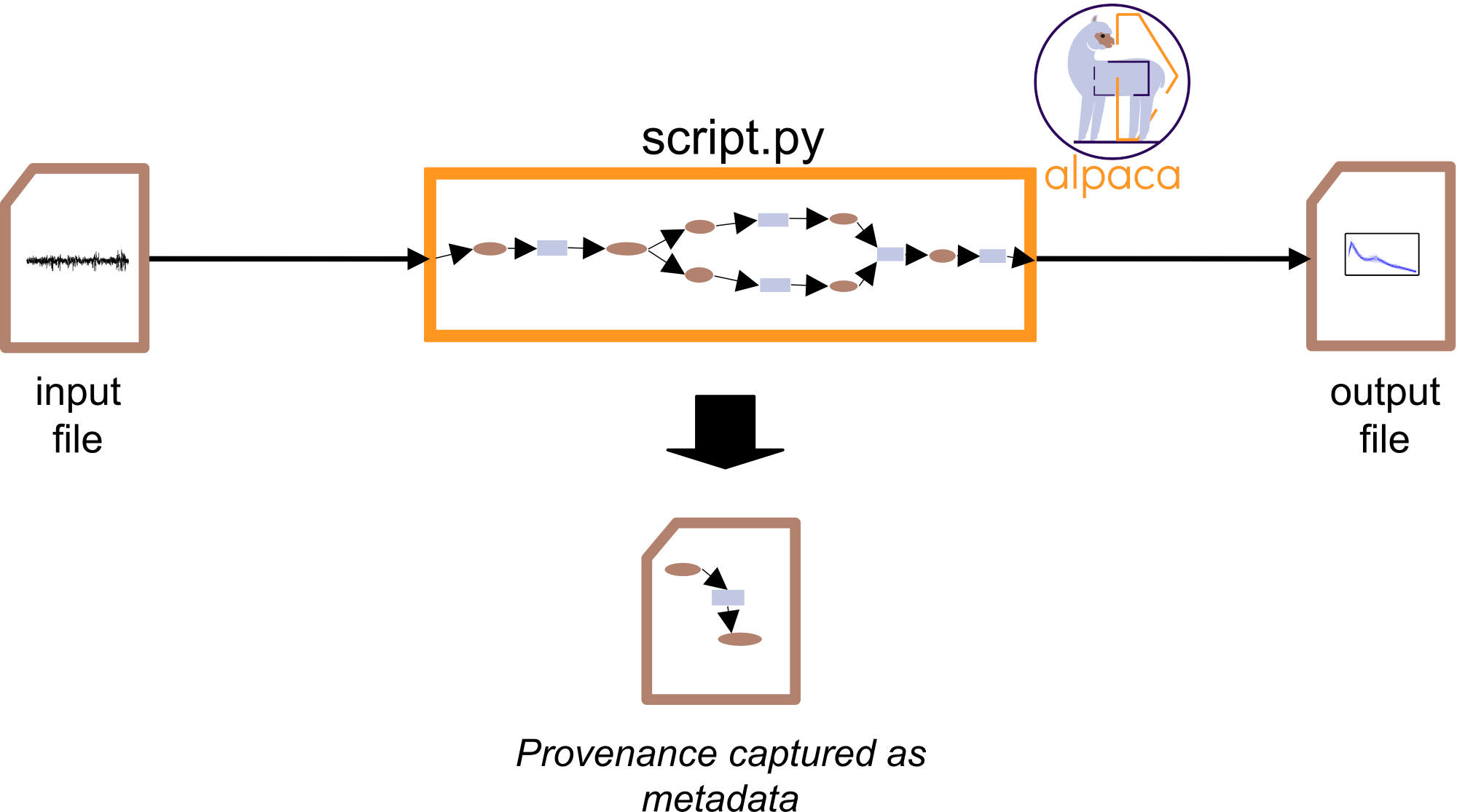

Supplement: Extended Data 1 — Download Extended Data 1, ZIP file. [file eneuro-11-ENEURO.0476-23.2024-s001.zip › doc/_static/images/alpaca/alpaca_overview.png]

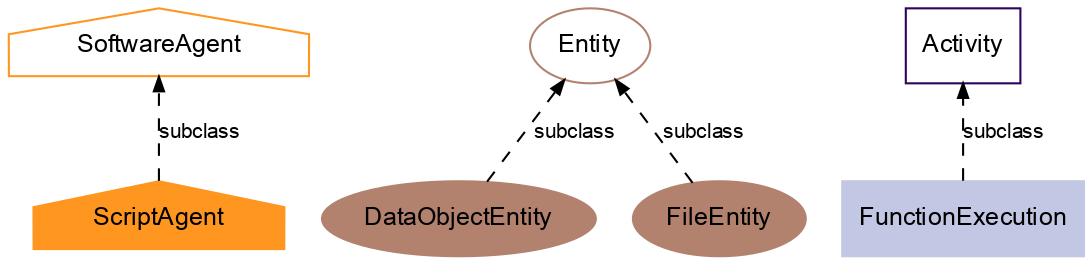

Supplement: Extended Data 1 — Download Extended Data 1, ZIP file. [file eneuro-11-ENEURO.0476-23.2024-s001.zip › doc/_static/images/alpaca/alpaca_prov.png]

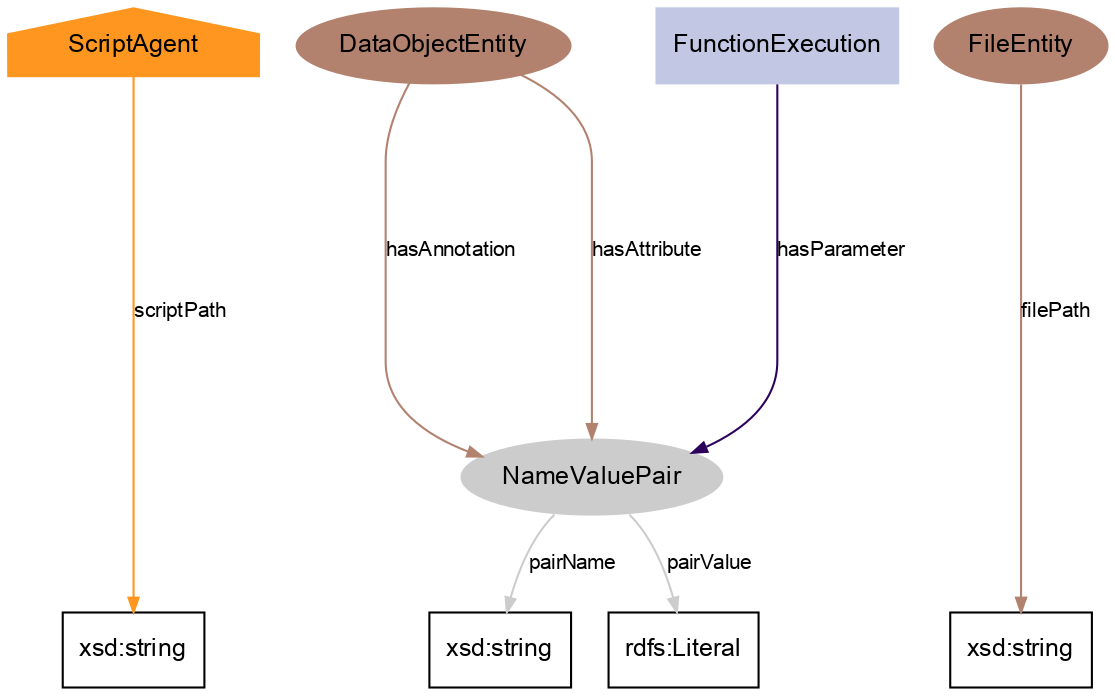

Supplement: Extended Data 1 — Download Extended Data 1, ZIP file. [file eneuro-11-ENEURO.0476-23.2024-s001.zip › doc/_static/images/alpaca/alpaca_prov_properties.png]

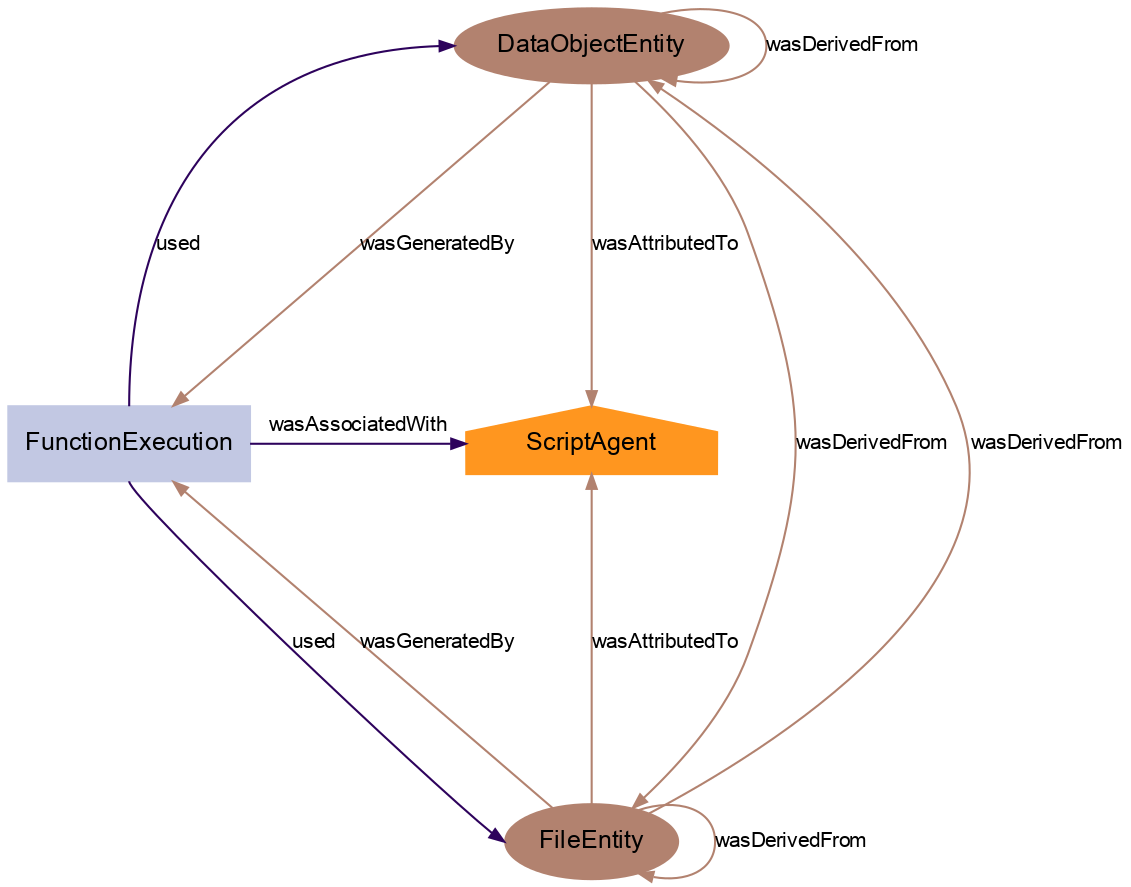

Supplement: Extended Data 1 — Download Extended Data 1, ZIP file. [file eneuro-11-ENEURO.0476-23.2024-s001.zip › doc/_static/images/alpaca/alpaca_prov_relationships.png]

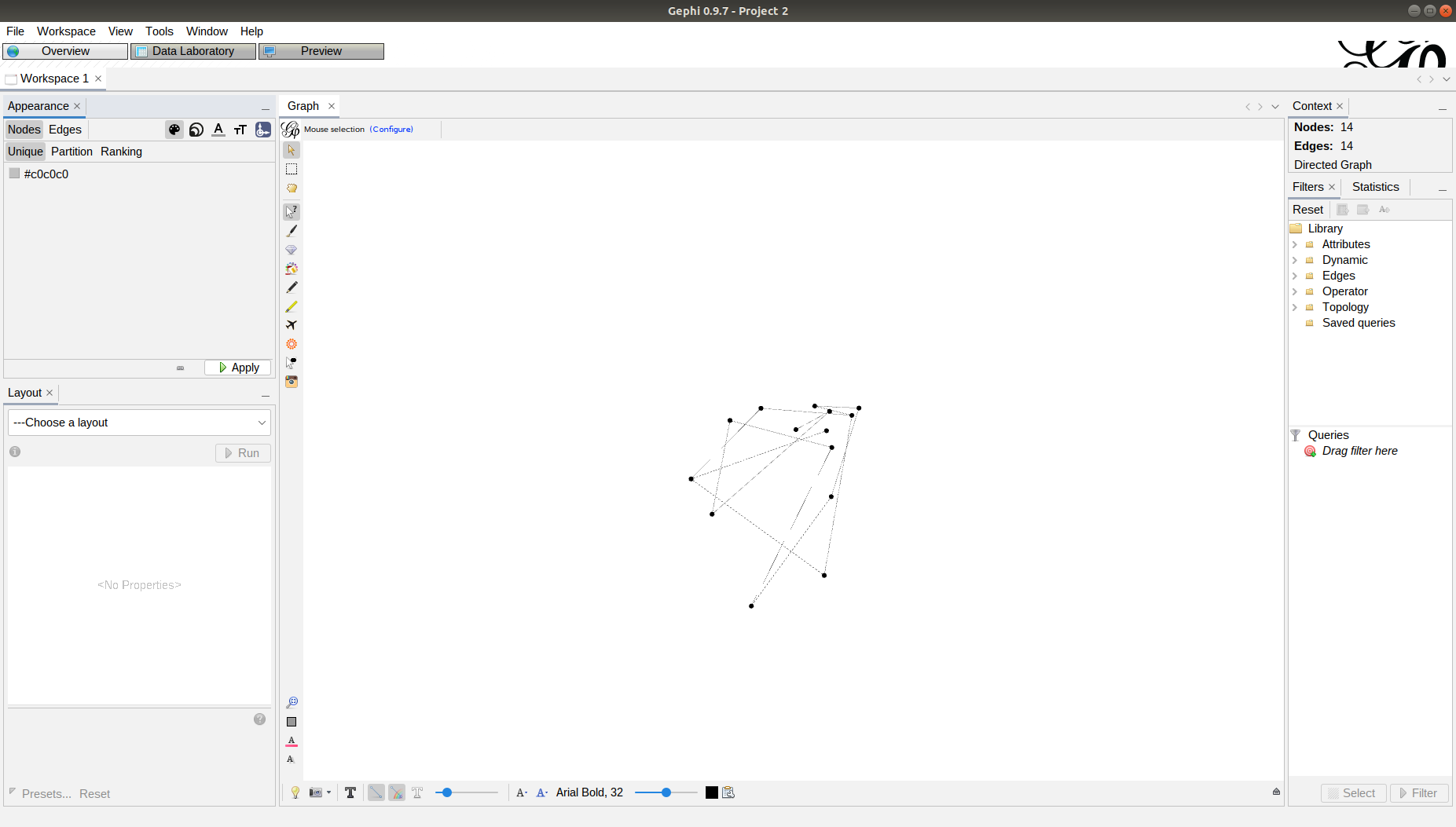

Supplement: Extended Data 1 — Download Extended Data 1, ZIP file. [file eneuro-11-ENEURO.0476-23.2024-s001.zip › doc/_static/images/gephi/screenshot_1.png]

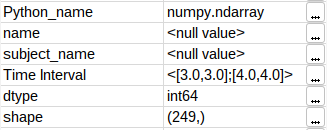

Supplement: Extended Data 1 — Download Extended Data 1, ZIP file. [file eneuro-11-ENEURO.0476-23.2024-s001.zip › doc/_static/images/gephi/screenshot_10.png]

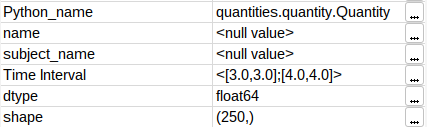

Supplement: Extended Data 1 — Download Extended Data 1, ZIP file. [file eneuro-11-ENEURO.0476-23.2024-s001.zip › doc/_static/images/gephi/screenshot_11.png]

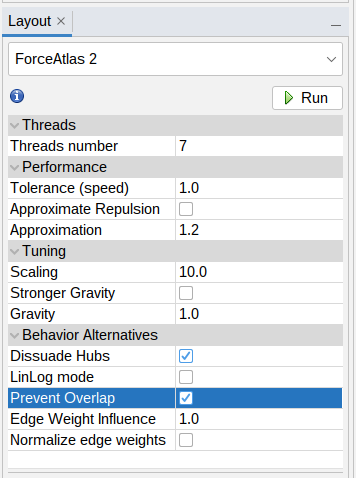

Supplement: Extended Data 1 — Download Extended Data 1, ZIP file. [file eneuro-11-ENEURO.0476-23.2024-s001.zip › doc/_static/images/gephi/screenshot_2.png]

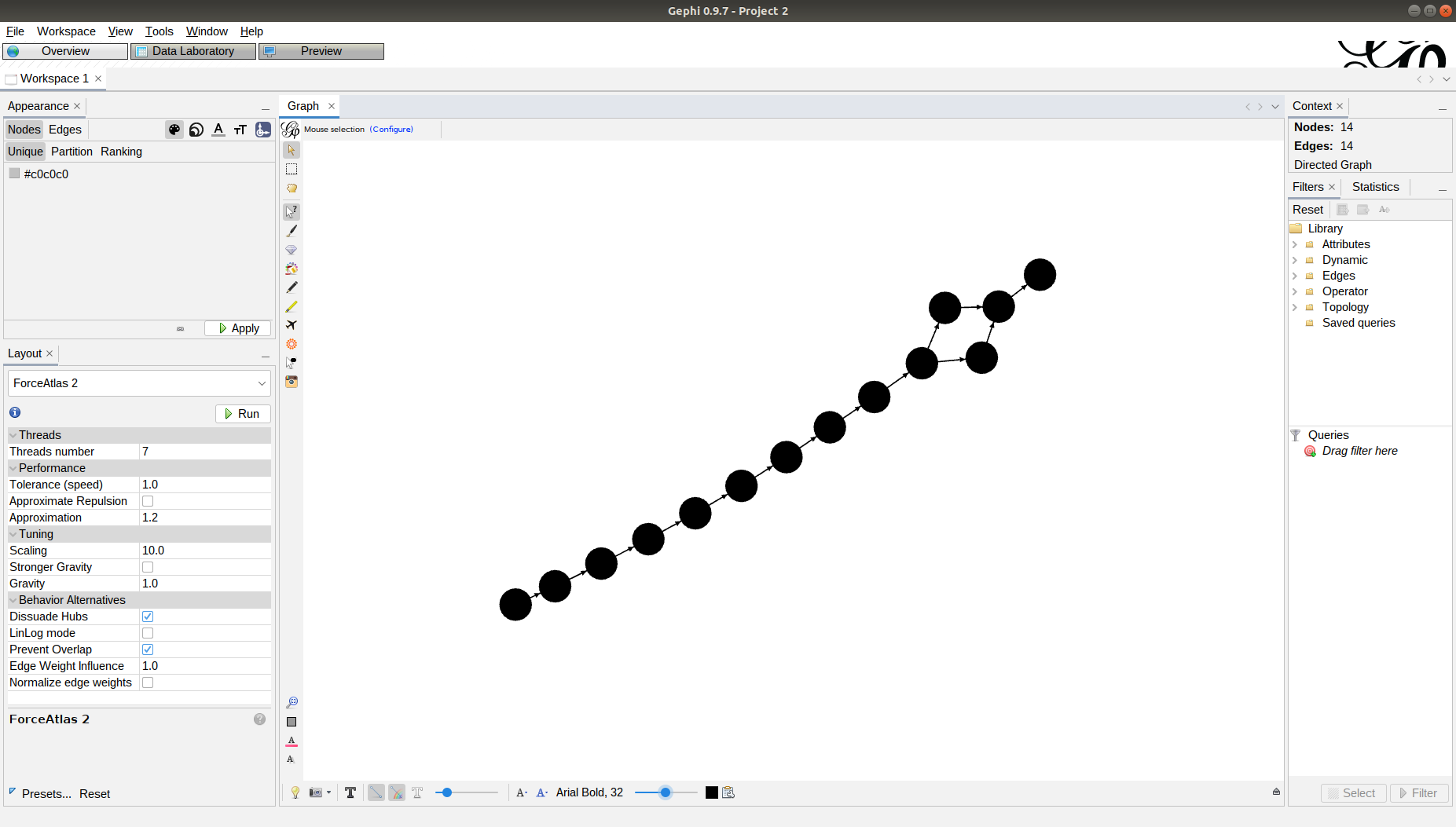

Supplement: Extended Data 1 — Download Extended Data 1, ZIP file. [file eneuro-11-ENEURO.0476-23.2024-s001.zip › doc/_static/images/gephi/screenshot_3.png]

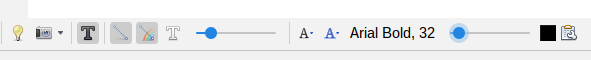

Supplement: Extended Data 1 — Download Extended Data 1, ZIP file. [file eneuro-11-ENEURO.0476-23.2024-s001.zip › doc/_static/images/gephi/screenshot_4.png]

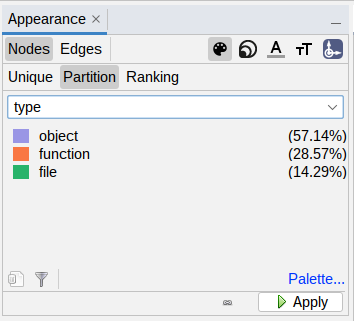

Supplement: Extended Data 1 — Download Extended Data 1, ZIP file. [file eneuro-11-ENEURO.0476-23.2024-s001.zip › doc/_static/images/gephi/screenshot_5.png]

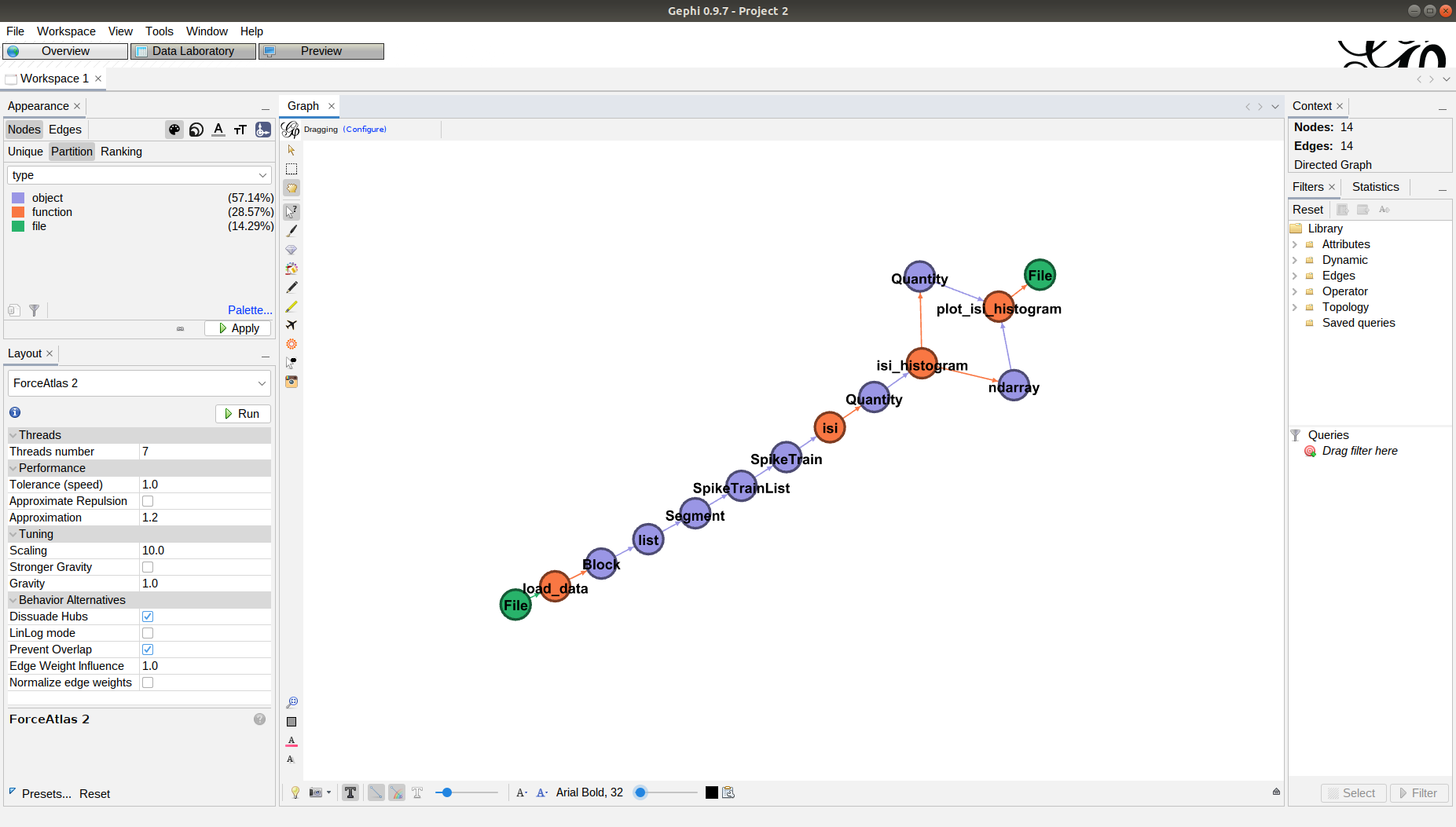

Supplement: Extended Data 1 — Download Extended Data 1, ZIP file. [file eneuro-11-ENEURO.0476-23.2024-s001.zip › doc/_static/images/gephi/screenshot_6.png]

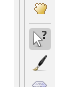

Supplement: Extended Data 1 — Download Extended Data 1, ZIP file. [file eneuro-11-ENEURO.0476-23.2024-s001.zip › doc/_static/images/gephi/screenshot_7.png]

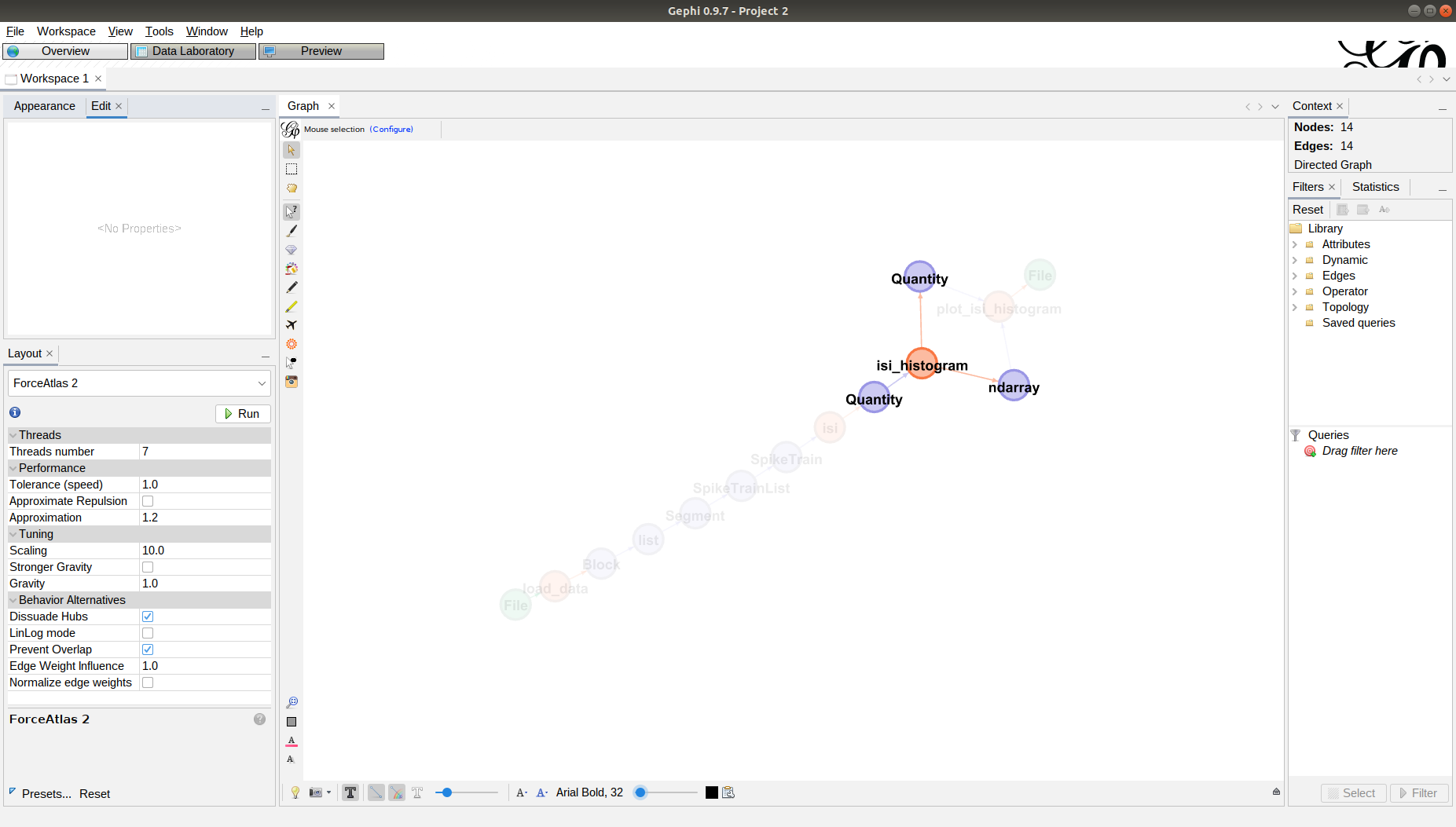

Supplement: Extended Data 1 — Download Extended Data 1, ZIP file. [file eneuro-11-ENEURO.0476-23.2024-s001.zip › doc/_static/images/gephi/screenshot_8.png]

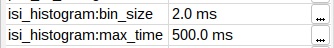

Supplement: Extended Data 1 — Download Extended Data 1, ZIP file. [file eneuro-11-ENEURO.0476-23.2024-s001.zip › doc/_static/images/gephi/screenshot_9.png]

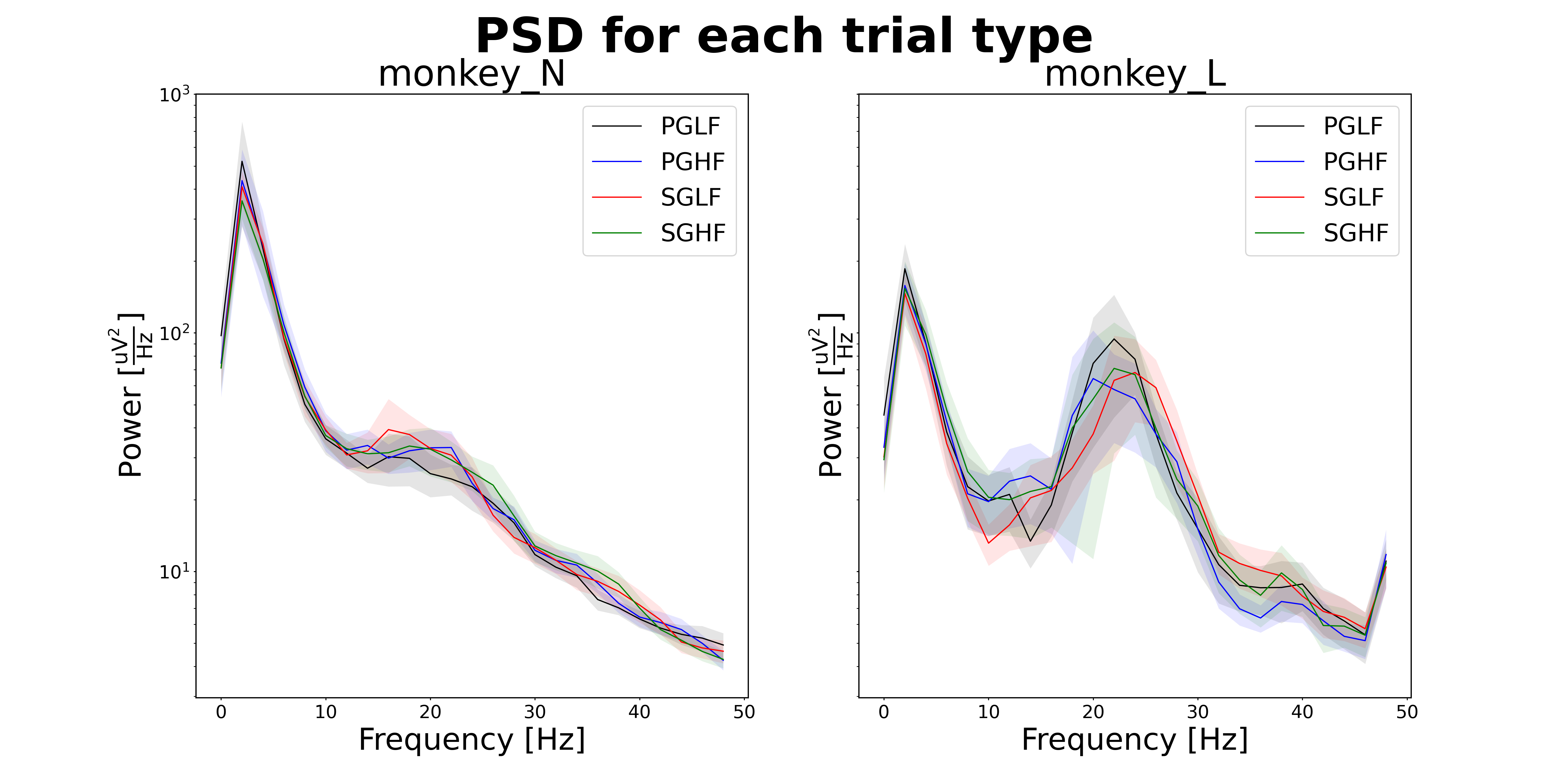

Supplement: Extended Data 2 — Download Extended Data 2, ZIP file. [file eneuro-11-ENEURO.0476-23.2024-s002.zip › outputs/no_provenance/R2G_PSD_all_subjects.png]

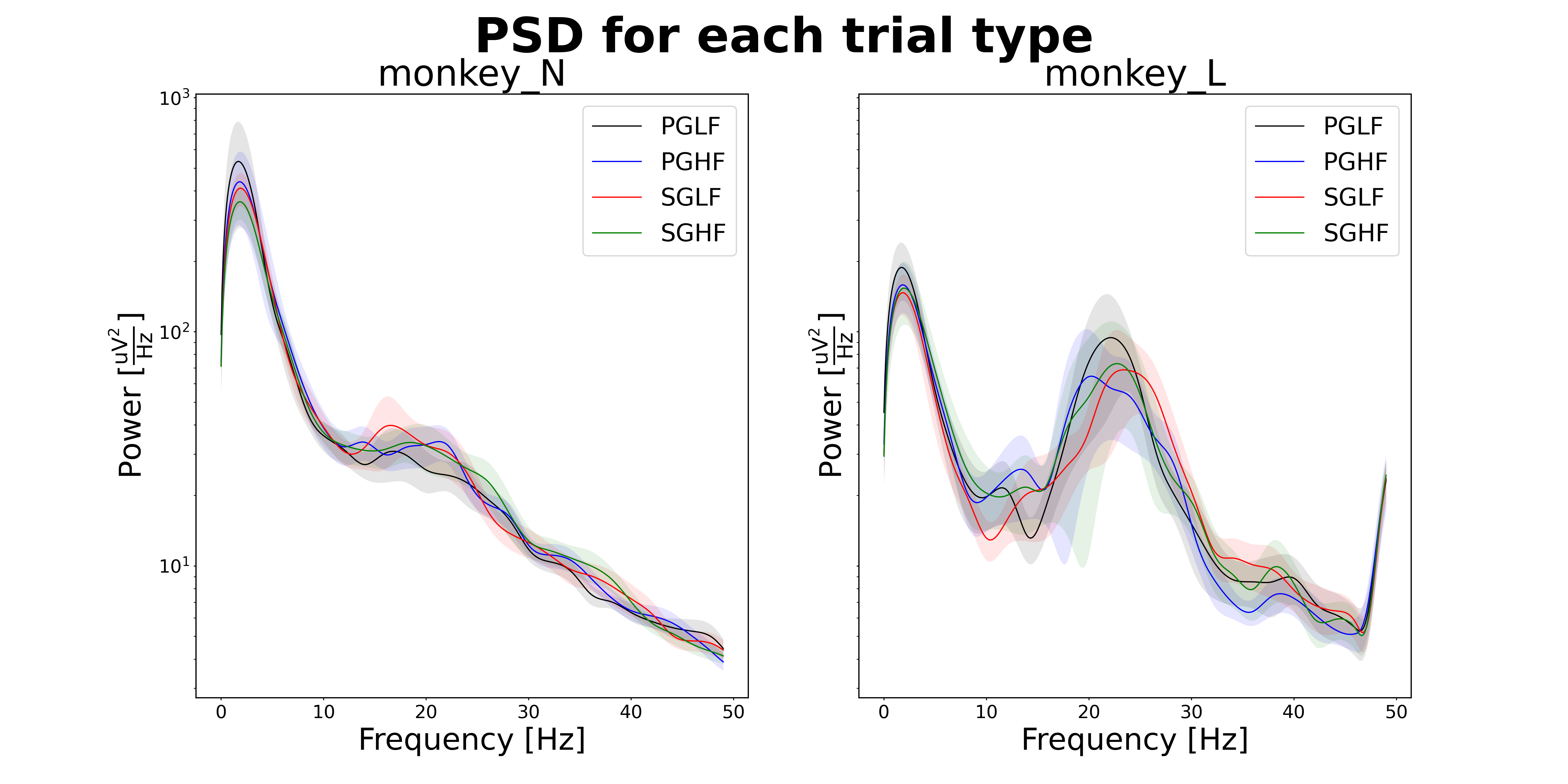

Supplement: Extended Data 2 — Download Extended Data 2, ZIP file. [file eneuro-11-ENEURO.0476-23.2024-s002.zip › outputs/smoothed_plot/R2G_PSD_all_subjects.png]
