# Supplementary material for: Facilitating the Sharing of Electrophysiology Data Analysis Results Through In-Depth Provenance Capture
Source: eNeuro. 2024 Jun 7;11(6):ENEURO.0476-23.2024. doi: 10.1523/ENEURO.0476-23.2024 (PMC11181106; doi:10.1523/ENEURO.0476-23.2024)
Supplement: Extended Data 2 — Download Extended Data 2, ZIP file. [file eneuro-11-ENEURO.0476-23.2024-s002.zip › documents/code_changes.pdf]

# Changes to the code necessary to track provenance using Alpaca

The script *psd_by_trial_type.py* was modified to:

- apply the decorator to functions imported from other modules or to methods inside classes defined in other modules;
- apply the decorator to user-defined functions in the script (i.e., using the Python *def* keyword);
- activate provenance tracking at the beginning of the *main* function;
- serialize the provenance trace as a Turtle file at the end of the *main* function.

This output is produced by running *git diff –no-index ./code/no_provenance/psd_by_trial_type.py ./code/provenance/psd_by_trial_type.py*, which are the versions of *psd_by_trial_type.py* without and with Alpaca provenance tracking, respectively. These scripts are available in this archive.

```
diff --git a/./code/no_provenance/psd_by_trial_type.py b/./code/provenance/psd_by_trial_type.py
index 6bc7f5d..f45e70e 100644
--- a/./code/no_provenance/psd_by_trial_type.py
+++ b/./code/provenance/psd_by_trial_type.py
@@ -17,12 +17,37 @@ import matplotlib.pyplot as plt
 from numpy import mean
 from scipy.stats import sem

+from alpaca import Provenance, activate, save_provenance, alpaca_setting
+from alpaca.utils.files import get_file_name
+
+
+# Apply the decorator to the functions used
+
+butter = Provenance(inputs=[’signal’])(butter)
+
+welch_psd = Provenance(inputs=[’signal’])(welch_psd)
+
+add_epoch = Provenance(inputs=[’segment’, ’event1’, ’event2’])(add_epoch)
+
+get_events = Provenance(inputs=[’container’],
+                       container_output=True)(get_events)
+
+cut_segment_by_epoch = Provenance(inputs=[’seg’, ’epoch’],
+                                 container_output=True)(cut_segment_by_epoch)
+
+mean = Provenance(inputs=[’a’])(mean)
+
+sem = Provenance(inputs=[’a’])(sem)
+
+neo.AnalogSignal.downsample = Provenance(inputs=[’self’])(neo.AnalogSignal.downsample)
+

 # Setup logging
```

```
 logging.basicConfig(level=logging.INFO,
                     format="[%(asctime)s] %(name)s %(levelname)s: %(message)s")


+@Provenance(inputs=[], file_input=['file_name'])
 def load_data(file_name):
     """
     Reads all blocks in the NIX data file `file_name`.
@@ -33,6 +58,7 @@ def load_data(file_name):
     return block


+@Provenance(inputs=[])
 def create_main_plot_objects(n_subjects, title):
     """
     Creates the plotting grid and figure/axes, with proper distribution and
@@ -62,6 +88,7 @@ def create_main_plot_objects(n_subjects, title):
     return fig, axes


+@Provenance(inputs=['signal'])
 def select_channels(signal, skip_channels):
     """
     Selects all the channels from the AnalogSignal `signal` that are not
@@ -75,6 +102,7 @@ def select_channels(signal, skip_channels):
     return signal[:, mask]


+@Provenance(inputs=['axes', 'freqs', 'psds', 'sem'])
 def plot_lfp_psd(axes, freqs, psds, sem, label, sem_multiplier=1.96,
                  freq_range=None, **kwargs):
     """
@@ -99,6 +127,7 @@ def plot_lfp_psd(axes, freqs, psds, sem, label, sem_multiplier=1.96,
     return axes


+@Provenance(inputs=['axes', 'title'])
 def set_title(axes, title):
     """
     Set the title of the plot in `axes`.
@@ -108,6 +137,7 @@ def set_title(axes, title):
     axes.set_title(title)


+@Provenance(inputs=['fig'], file_output=['file_name'])
 def save_plot(fig, file_name, **kwargs):
     """
     Save the plot in `fig` to the file `file_name`.
@@ -115,6 +145,7 @@ def save_plot(fig, file_name, **kwargs):
     fig.savefig(file_name, **kwargs)


+@Provenance(inputs=['arrays'])
 def vstack_quantities(*arrays):
     """
     Performs stacking of Quantity arrays, as ordinary `np.vstack` removes
@@ -144,6 +175,14 @@ def vstack_quantities(*arrays):
```

```
 def main(session_files, output_dir, skip_channels):
+
+    # Use builtin hash for matplotlib objects
+    alpaca_setting('use_builtin_hash_for_module', ['matplotlib'])
+    alpaca_setting('authority', "fz-juelich.de")
+
+    # Activate provenance tracking
+    activate()
+
     logging.info(f"Processing files: ','.join(session_files)")

     # Labels of the different trial types in the data, and the colors
@@ -236,6 +275,11 @@ def main(session_files, output_dir, skip_channels):
     logging.info(f"Saving output to out_file")
     save_plot(fig, out_file, format='png', dpi=300)

+    # Save provenance information as Turtle file
+    prov_file_format = "ttl"
+    prov_file = get_file_name(out_file, extension=prov_file_format)
+    save_provenance(prov_file, file_format=prov_file_format)
+

 if __name__ == "__main__":
     parser = argparse.ArgumentParser()
```
